# Humic Acid Enhances the Growth of Tomato Promoted by Endophytic Bacterial Strains Through the Activation of Hormone-, Growth-, and Transcription-Related Processes

**DOI:** 10.3389/fpls.2020.582267

**Published:** 2020-09-16

**Authors:** Nikoletta Galambos, Stéphane Compant, Marco Moretto, Carmela Sicher, Gerardo Puopolo, Felix Wäckers, Angela Sessitsch, Ilaria Pertot, Michele Perazzolli

**Affiliations:** ^1^ Research and Innovation Centre, Fondazione Edmund Mach, San Michele all’Adige, Italy; ^2^ Department of Civil, Environmental and Mechanical Engineering, University of Trento, Trento, Italy; ^3^ Biobest NV, Westerlo, Belgium; ^4^ Center for Health and Bioresources, AIT Austrian Institute of Technology, Tulln, Austria; ^5^ Center Agriculture Food Environment (C3A), University of Trento, San Michele all’Adige, Italy

**Keywords:** plant growth-promoting bacterial endophytes, humic acid, transcriptomic, RNA sequencing, tomato, endophytes, plant growth promoting rhizobacteria

## Abstract

Plant growth-promoting bacteria (PGPB) are promising alternatives in the reduction of the use of chemical fertilizers. Likewise, humic acid (HA) can improve plant growth and/or the establishment of endophytic PGPB. Although the effects of PGPB colonization or HA treatment have been studied separately, little information is available on plant response to the combined applications of PGPB and HA. Thus, the aim of this work was to understand the physiological effects, bacterial colonization and transcriptional responses activated by endophytic bacterial strains in tomato roots and shoots in the absence (control condition) and presence of HA (HA condition). Tomato shoot length was promoted by seed inoculation with *Paraburkholderia phytofirmans* PsJN, *Pantoea agglomerans* D7G, or *Enterobacter* sp. 32A in the presence of HA, indicating a possible complementation of PGPB and HA effects. Tomato colonization by endophytic bacterial strains was comparable in the control and HA condition. The main transcriptional regulations occurred in tomato roots and the majority of differentially expressed genes (DEGs) was upregulated by endophytic bacterial strains in the HA condition. Half of the DEGs was modulated by two or three strains as possible common reactions to endophytic bacterial strains, involving protein metabolism, transcription, transport, signal transduction, and defense. Moreover, strain-specific tomato responses included the upregulation of signal transduction, transcription, hormone metabolism, protein metabolism, secondary metabolism, and defense processes, highlighting specific traits of the endophyte-tomato interaction. The presence of HA enhanced the upregulation of genes related to signal transduction, hormone metabolism, transcription, protein metabolism, transport, defense, and growth-related processes in terms of number of involved genes and fold change values. This study provides detailed information on HA-dependent enhancement of growth-related processes stimulated by endophytic bacterial strains in tomato plants and reports the optimized dosages, complementation properties and gene markers for the further development of efficient PGPB- and HA-based biostimulants.

## Introduction

Conventional agriculture largely depends on chemical fertilizers (e.g., nitrogen-, phosphorus-, potassium-, and micro element-based fertilizers), which have numerous environmental drawbacks, such as surface and groundwater pollution and denitrification processes (Khan et al., 2018). Among crop plants, tomato (*Solanum lycopersicum*) is cultivated worldwide under field and greenhouse conditions (Hobson and Grierson, 1993) and requires an extensive use of chemical fertilizers that cause a significant negative environmental impact (Maham et al., 2020).

Plant growth-promoting bacteria (PGPB) can improve plant development and increase nutrient supply, such as nitrogen and iron (Ferreira et al., 2019). PGPB application has been considered as a promising alternative to maintain agroecosystem health and productivity (Gouda et al., 2018). Some PGPB can colonize the internal tissues of numerous plant species (endophytes) and can positively influence plant growth through various mechanisms, including the production of hormones, the improvement of nutrient uptake and protection against biotic or abiotic stresses (Gaiero et al., 2013). In particular, species of the bacterial genera *Bacillus*, *Enterobacter*, *Microbacterium*, *Pantoea*, *Paraburkholderia* and *Sphingomonas* are known to establish this type of association with plants (Sessitsch et al., 2005; Campisano et al., 2014; Hardoim et al., 2015). For example, bacterial endophytes isolated from grapevine, such as *Microbacterium* sp. C9D (C9D), *Pantoea agglomerans* D7G (D7G), *P. eucalypti* 727 (727), and *Sphingomonas* sp. 11E (11E), were able to increase the seed germination of *Arabidopsis thaliana* and exhibited beneficial traits *in vitro*, such as 1-aminocyclopropane-1-carboxylic acid (ACC)-deaminase activity (Campisano et al., 2014; Lòpez-Fernàndez et al., 2015a). Other endophytic bacteria, such as *Bacillus* sp. 54A (54A) and *Enterobacter* sp. 32A (32A), inhibited the growth of plant pathogens (e.g., *Botrytis cinerea*, *Botryosphaeria dothidea*, and *Botryosphaeria obtusa*) in dual-culture plate tests, suggesting that these strains can potentially protect plants against infections (Campisano et al., 2014; Lòpez-Fernàndez et al., 2015b). Among them, 32A affected the secondary metabolism and activated possible defense pathways in grapevine (Lòpez-Fernàndez et al., 2015a). A widely studied plant endophyte, *Paraburkholderia phytofirmans* PsJN (PsJN), previously classified as *Pseudomonas* and *Burkholderia* genus (Sessitsch et al., 2005; Sawana et al., 2014) is known to increase *A. thaliana* tolerance to salt stress through transcriptional and metabolic changes, such as proline accumulation, abscisic acid signaling and reactive oxygen species (ROS) scavenging (Pinedo et al., 2015). In particular, PsJN is able to improve the growth (Pillay and Nowak, 1997; Sharma and Nowak, 1998) and heat tolerance (Issa et al., 2018) of tomato plants, increasing net photosynthesis rate, stomatal conductance and chlorophyll content. For these reasons, the use of PGPB could be a promising approach in tomato production to improve plant growth and to reduce the use of chemical fertilizers. However, limitations to the wide use of beneficial endophytes were often encountered, for example because of the variable and/or inconsistent effect on the plant, especially under field conditions (Martínez-Viveros et al., 2010; Timmusk et al., 2017). Although they have been relatively well studied, a better understanding on the bacterial colonization (e.g., colonization rate and stability, competition with other microorganisms) and effects on tomato physiology (e.g., transcriptional response) is needed, in order to develop more efficient PGPB-based biofertilizers.

In addition to PGPB, organic humic substances present in the soil [e.g., humic acid (HA), humin and fulvic acid] can also improve plant growth and health and act as biostimulants (Olivares et al., 2017). Biostimulants are organic bioactive compounds that affect plant metabolism (Drobek et al., 2019). Among the natural biostimulants, HA is abundant in soil, peat or lignite and derives from the decay of organic materials (Drobek et al., 2019). HA improves nutrient uptake and the growth of tomato plants under hydroponic (Adani et al., 1998) and greenhouse conditions (Dursun et al., 2002), increasing electrolyte leakage, cell permeability, and nutrient accumulation (David et al., 1994). HA is a mixture of polymeric organic compounds, stabilized by weak forces (hydrophobic and hydrogen bonds) in a supramolecular arrangement that forms hydrophobic domains (Fischer, 2017). HA is refractory to degradation and its hydrophobic domains can provide protection for selected PGPB (Piccolo, 1996; Canellas and Olivares, 2014). The hydrophobic HA domain undergoes conformational changes in the presence of organic acids derived from root exudates and releases PGPB for the interaction with host plants (Nardi et al., 2009; Olivares et al., 2017). HA can also contribute to the endophytic establishment of PGPB (Olivares et al., 2017) and it has been suggested as a suitable carrier for PGPB formulation (Young et al., 2006; Olivares et al., 2017; Ma, 2019). For example, *Herbaspirillium seropedicae* Z67 inoculation in the presence of HA increased root surface area, enhanced grain production and altered carbohydrate and nitrogen metabolism in maize plants (Canellas et al., 2012). In particular, in low fertility soils, *H. seropedicae* Z67 and HA increased maize production compared to non-inoculated plants through PGPB-driven hormone production and HA-stimulated changes in phenolic metabolism (Canellas et al., 2015). Likewise, tomato fruit biomass was increased by *H. seropedicae* HRC54 and HA through the stimulation of nitrogen and secondary metabolism (Olivares et al., 2015). A mixed inoculum of *H. seropedicae* HRC54 and *Gluconacetobacter diazotrophicus* PAL 5 in combination with HA changed the metabolite fingerprints of amino acids, sugars and organic acids in maize and sugarcane seedlings, indicating that the activation of primary and secondary metabolism was partially responsible for the biostimulation effects (Aguiar et al., 2018; Canellas et al., 2019). Although considerable evidence of efficacy exist in literature, the molecular mechanisms of the combined applications of living PGPB and organic biostimulant on crops are less investigated (Bulgari et al., 2015). Our goal was to improve the understanding of the complementation effects and cellular pathways activated by endophytic bacterial strains and HA for the further development of sustainable biofertilizers for tomato production. More specifically, the present study aimed at understanding the colonization, growth promotion effects and transcriptional responses in tomato plants inoculated with bacterial endophytes in the absence (control condition) and presence of HA (HA condition).

## Material and Methods

### Growth of Bacterial Strains and Inoculum Preparation

The bacterial strains *Microbacterium* sp. C9D (C9D; isolate MiVv2), *Bacillus* sp. 54A (54A; isolate BaVs16), *Pantoea eucalypti* 727 (727; isolate PaVv9), *Pantoea agglomerans* D7G (D7G; isolate PaVv7), *Enterobacter* sp. 32A (32A; isolate EnVs6), and *Sphingomonas* sp. 11E (11E) were previously isolated from the grapevine endosphere (Campisano et al., 2014), while *Paraburkholderia phytofirmans* PsJN (PsJN) was isolated from surface-sterilized onion roots (Sessitsch et al., 2005). Bacterial strains were stored in 80% glycerol at −80°C and were grown in 5-ml nutrient broth (NB) in sterile 15-ml tubes at 25°C for 24 h under orbital shaking at 220 rpm.

For seed inoculation, bacterial cells were collected by centrifugation at 3,500 g for 10 min and washed twice with sterile 10 mM MgSO_4_. Bacterial cells were then suspended in sterile 10 mM MgSO_4_ and the bacterial suspension was adjusted to 1.0 × 10^7^ colony forming units (CFU) per unit of volume (CFU ml^−1^) based on an optical density conversion table at 600 nm (OD_600_) optimized for each strain (**Table S1**).

Since HA is poorly soluble in water, a stock solution (1 g L^−1^) of HA (Sigma-Aldrich, St. Louis, Missouri, USA; code 53680) was prepared in 0.1 M NaOH and the pH was then adjusted to 6.8 with 70% HNO_3_ (HA stock solution) to avoid acidification of the NB and half-strength Hoagland. Since NaNO_3_ was formed in the HA preparation, a water solution with NaOH and HNO_3_ at an equivalent concentration to the HA stock solution was used as control in the bacterial compatibility, tomato seed inoculation, and transcriptomic analyses (control stock solution).

### Bacterial Compatibility Assay With Humic Acid

To assess the bacterial compatibility with HA, 20 µl of each bacterial suspension (1.0 × 10^7^ CFU ml^−1^) was inoculated in 200 µl NB supplemented with 50 mg L^−1^ HA (10 µl HA stock solution) in a 96-well microplate (Thermo Fisher Scientific, Waltham, MA, USA). NB supplemented with 10 µl control stock solution was used as control (0 mg L^−1^ HA). Samples were incubated at 25°C for 72 h under orbital shaking programmed at medium shaking speed and bacterial growth was monitored by measuring the OD_600_ every 30 min using a Synergy 2 Multi-Mode Microplate Reader (Biotek, Winooski, VT, USA). Six replicates (wells) were used for each treatment and the experiment was carried out twice.

### Tomato Seed Inoculation and Growth Conditions in Glass Tube and Square Dish

Seeds of *S. lycopersicum* L. cv. Moneymaker (Justseed, Wrexham, UK) were treated with 70% ethanol for 1 min and 2% sodium hypochlorite containing 0.02% Tween 20 for 5 min in a 50 ml tube (Subramanian et al., 2015) with vigorous shaking and washed three times with sterile distilled water (3 min each), in order to reduce the number of seed-associated microorganisms. Surface-sterilized seeds (50 seeds) were treated with 5 ml of sterile 10 mM MgSO_4_ (mock-inoculated) or inoculated with 5 ml of the bacterial suspension (bacterium-inoculated) of the respective endophytic strain (1 × 10^7^ CFU ml^−1^) by overnight incubation at 25 ± 1°C in a sterile 15-ml tube under orbital shaking at 40 rpm. Seeds were transferred to Petri dishes (20 seeds for each dish) containing 1% water agar (Thermo Fisher Scientific) and incubated for 48 h in a growth chamber (Binder KBWF 720, Bohemina, NY, USA) at 25 ± 1°C with a 16 h photoperiod (photon flux density of 0.033 mmol s^−1^ m^−2^) to allow seed germination.

Germinated seeds with the same root length (1 mm) were selected and transferred to the growth medium in a glass tube or in a square dish as described below. To optimize the HA concentration for tomato plants, each germinated seed was transferred into a sterile 95 ml glass tube (Artiglass, Padova, Italy) containing 2.5 g sterile perlite and 10-ml half-strength Hoagland with 0, 25, 50, or 100 mg L^−1^ HA, and incubated in the growth chamber for six weeks. To assess the effect of HA on bacterium-inoculated plants, five seeds were transferred along a line in a central position of a 10 cm square dish (Sarstedt, Nümbrecht, Germany) containing 50 ml solid (14 g L^−1^ agar) half-strength Hoagland with 0 mg L^−1^ (control condition; 2.5 ml control stock solution for each dish) or 50 mg L^−1^ HA (HA condition; 2.5 ml HA stock solution for each dish), as optimized HA concentration. Dishes were incubated in vertical position in the growth chamber, shoot and root length was measured with a ruler and the fresh weight of the whole plant was assessed with a precision balance at three and six days after incubation (DAI). Four and five replicates were analyzed for each treatment in the experiment with glass tubes and square dishes, respectively, and each experiment was carried out twice.

### Bacterial Re-Isolation From Tomato Plants

At the end of the incubation period, mock-inoculated and bacterium-inoculated plants were collected, and each plant was surface-sterilized in a 50 ml tube with 70% ethanol for 1 min, 2% sodium hypochlorite for 1.5 min, followed by 70% ethanol for 1 min. Plants were washed three times with distilled water (2 min each), dried with a sterile filter paper before the assessment of the fresh weight. Plants were ground in a mixer-mill disruptor (MM 400, Retsch, Haan, Germany) at 25 Hz for 2 min in presence of 500 µl potassium phosphate buffer (1 mM, pH 7). Each suspension was serially diluted and 10 µl aliquots were plated in triplicates on nutrient agar (NA). Aliquots (10 µl) of the last washing solution were plated as the control of surface sterilization. After incubation at 25°C for 3 days, CFU values of endophytic bacterial strains per unit of plant fresh weight (CFU g^−1^) were calculated. Five replicates (plants) were analyzed for each treatment and the experiment was carried out twice.

### Fluorescence *In Situ* Hybridization Using Double Labeling of Oligonucleotide Probes

Double labeling of oligonucleotide probes for fluorescence *in situ* hybridization (DOPE-FISH) was performed on mock-inoculated plants and PsJN-, D7G-, or 32A-inoculated plants at 3 and 6 DAI in the control or HA condition in square dishes. Plants were aseptically cut into roots, stem, and leaves and were sectioned transversally using razor blades. Samples were then fixed in a 4% paraformaldehyde in 1× phosphate-buffered saline (PBS) solution at 4°C for 5 h and were rinsed three times with 1× PBS as previously reported (Compant et al., 2011). Plants were dehydrated in increasing concentrations of ethanol solution (25%, 50%, 75%, and 99%; 20 min each step) and stored at 4°C. DOPE-FISH was carried out using probes from Eurofins (Germany) labeled at both the 5’ and 3’ positions. A probe mixture targeting eubacteria, composed of EUB338, EUB338II, EUB338III (EUBmix) coupled with a Cy3 fluorochrome and Bphyt probe targeting the 23S rRNA gene of PsJN coupled with Cy5 (Amann et al., 1990; Daims et al., 1999; Mitter et al., 2017). For D7G and 32A, EUBmix and Gam42a probe targeting the 23S rRNA gene of D7G and 32A coupled with Cy5 was used (Manz et al., 1992). NONEUB probe coupled with Cy3 or Cy5 was used independently as negative control (Wallner et al., 1993). Fluorescent *in situ* hybridization was carried out in sterile 1.5 ml tubes at 46°C for 2 h in the dark with 60 µl hybridization buffer for PsJN (containing 0.9 M NaCl; 0.02 M Tris HCl, 0.01% SDS, 10% formamide and 5 ng µl^−1^ of each probe) and with 60 µl hybridization buffer for D7G and 32A (containing 0.9 M NaCl; 0.02 M Tris HCl, 0.01% SDS, 35% formamide, and 5 ng µl^−1^ of each probe). Washing was conducted at 48°C for 30 min with a pre-warmed post-FISH solution containing 0.02 M Tris HCl, 0.01% SDS, NaCl and EDTA at a concentration corresponding to the formamide concentration. Samples were then rinsed with distilled water before overnight air-drying in the dark. Samples were observed under a confocal microscope (Olympus Fluoview FV1000 with multiline laser FV5-LAMAR-2 and HeNe (G) laser FV10-LAHEG230-2). Pictures were taken at 405, 488, 633 nm wavelengths with Cy3 assigned as green and Cy5 as red. Pictures were analyzed using Imaris 8 software (BITPLANE, UK). Z-stacks were used to generate whole-stack pictures. Five replicates (plants) were analyzed for each treatment and representative pictures were selected. Pictures were cropped and light/contrast balance improved in post process.

### Sample Collection, RNA Extraction, and Illumina Sequencing

Mock-inoculated plants and PsJN-, D7G-, or 32A-inoculated plants in square dishes were collected at 3 DAI in the control and HA condition in square dishes. Five plants were randomly collected for each treatment (replicate), roots and shoots were cut, separately placed into 2 ml tubes, immediately frozen in liquid nitrogen and stored at −80°C. A metal bead was added to each tube and samples were ground in a mixer-mill disruptor (MM200, Retsch) at 25 Hz for 1 min. Total RNA was extracted from 0.1 g of ground sample using a Spectrum Plant Total RNA Kit (Sigma-Aldrich) with an on-column DNase treatment with RNase-Free DNase Set (Qiagen, Hilden, Germany). Total RNA was quantified using a Qubit (Thermo Fisher Scientific) and RNA quality was checked using a Tapestation 2200 (Agilent Technologies, Santa Clara, CA, USA). For each treatment, three replicates (pool of five plants) were analyzed. RNA samples were subjected to RNA-Seq library construction, using the TruSeq SBS v3 protocol (Illumina, SanDiego, CA, USA) and rRNA depletion with the RiboZero rRNA Removal Kit for plant according to the manufacturer’s instructions (Illumina). Paired-end reads of 150 nucleotides were obtained using a NovaSeq 6000 S2 instrument (Illumina) at the Institute of Applied Genomics (Udine, Italy) and sequences were deposited at the Sequence Read Archive of the National Center for Biotechnology (https://www.ncbi.nlm.nih.gov/sra) under the BioProject number PRJNA622763.

### Bioinformatic Analysis and Identification of Differentially Expressed Genes

Raw reads were cleaned and filtered using the programme Trimmomatic version 0.36 (Bolger et al., 2014) and low-quality bases with an average Phred quality score lower than 15 in a sliding window of four base were removed. Any resultant reads shorter than 36 bp in length were removed from the analysis and the quality check of filtered reads was performed using Fast QC version 0.11.7. Filtered read pairs were aligned and counted using STAR 2.7 (Dobin et al., 2013) to the *S. lycopersicum* genome release ITAG3.2 and counts of unambiguously mapped read pairs was obtained during the alignment with the STAR 2.7 program. Differentially expressed genes (DEGs) were identified with the Limma-Voom package (Law et al., 2014), which estimates the mean-variance relationship of Log_2_-transformed counts, generating a precision weight for each observation that is fed into the Limma empirical Bayes analysis pipeline (Smyth, 2006). A Volcano Plot was generated using the Python programming language and the matplotlib package (Hunter, 2007) and a double cut-off on *P*-value (*P* ≤ 0.01) and minimum Log_2_ fold change (FC) of one [Log_2_ (FC) ≥ 1 or Log_2_ (FC) ≤ −1] were imposed to identify DEGs through pairwise comparisons. Three pairwise comparisons were analyzed for shoots and roots: PsJN- vs. mock-inoculated, D7G- vs. mock-inoculated and 32A- vs. mock-inoculated plants. DEGs modulated by endophytic bacterial strains between the control and HA condition were compared in order to identify HA-dependent effects on processes activated by PsJN, D7G and 32A. Moreover, the pairwise comparison between the control and HA condition of mock-inoculated plants was included, in order to analyze the effects caused by HA in the absence of endophytic bacterial strains. The distribution of DEGs was summarized using the Venn diagram (http://bioinformatics.psb.ugent.be/webtools/Venn/) and DEGs were grouped in upregulated and downregulated genes by at least two endophytic bacterial strains (DEGs modulated by two or three strains), to highlight possible common reactions in response to endophytic bacterial strains, or specifically by only one endophytic bacterial strain (PsJN-, D7G-, or 32A-specific tomato DEGs) in the control and HA condition. The heat map diagram of fold change values of DEGs was visualized using the Java Treeview tool (Saldanha, 2004). Gene expression levels were then expressed as transcripts per million (TPM).

Gene Ontology (GO) terms and protein descriptions of tomato Heinz1706 genes (Sato et al., 2012) of the release ITAG3.2 were downloaded from the tomato genome browser (https://solgenomics.net/organism/Solanum_lycopersicum/genome). GO terms significantly overrepresented (*P* ≤ 0.05, Benjamin and Hochberg FDR correction) in the DEG lists in comparison to the whole tomato transcriptome were identified using the Biological Networks Gene Ontology (BiNGO) tool (Maere et al., 2005) and the biological networks were visualized with Cytoscape version version 3.7.1 (Shannon et al., 2003). DEGs were further annotated on the basis of tomato protein description and grouped into 14 functional categories according to the previous literature. Genes that were not associated to any biological process were assigned to the unknown function category. Tomato cellular pathways were generated with Biorender (https://biorender.com/) according to literature search of functional annotation of DEGs.

### Gene Expression Analysis by Quantitative Real-Time RT-PCR

Tomato gene markers were selected for quantitative real-time PCR (qPCR) analysis (**Table S2**). The ﬁrst strand of cDNA was synthesized from 1 μg of DNase-treated RNA using Superscript III (Invitrogen, Thermo Fisher Scientiﬁc) and oligo-dT primer. qPCR reactions were carried out with Platinum SYBR Green qPCR SuperMix-UDG (Invitrogen, Thermo Fisher Scientiﬁc) and speciﬁc primers using the Light Cycler 480 (Roche Diagnostics, Mannheim, Germany) as previously described (Perazzolli et al., 2016). Briefly, the PCR conditions were: 50°C for 2 min and 95°C for 2 min as initial steps, followed by 40 cycles of 95°C for 15 s and 60°C for 1 min. Each sample was examined in three technical replicates and dissociation curves were analyzed to verify the specificity of each amplification reaction. The Light Cycler 480 SV1.5.0 software (Roche) was used to extract Ct values based on the second derivative calculation and the LinReg software version 11.0 was used to calculate reaction efficiencies for each primer pair (Ruijter et al., 2009). For each gene, the relative expression level (fold change) was calculated according to the Pfaffl equation (Pfaffl, 2001) for each pairwise comparison between bacterium-inoculated and mock-inoculated samples in the control and HA condition. Five housekeeping genes were analyzed, such as genes encoding ankyrin repeat domain containing protein 2 (*ARD2*) (Pombo et al., 2017), kinesin light chain 2 isoform (*KLC*) (Pombo et al., 2017), vernalization insensitive 3 (*VIN3*) (Pombo et al., 2017), small nuclear ribonucleoprotein family protein (*LSM7*) (Müller et al., 2015), and ubiquitin carboxyl-terminal hydrolase (*UCH*) (Müller et al., 2015), and their stability was validated using the ΔCt method (Silver et al., 2006). *ARD2* was then selected as constitutive gene for normalization, because its expression was not affected by the different conditions (**Table S2**). Three replicates (pool of five plants) were analyzed for each condition.

### Statistical Analysis

All functional experiments were carried out twice and data were analyzed with the Past 3.26 software (Hammer et al., 2001). After validating data for normal distribution (Shapiro-Wilk test, *P* > 0.05) and variance homogeneity of the data (Levene’s tests, *P* > 0.05), each experimental repetition was analyzed singularly and a two-way analysis of variance (ANOVA) was used to demonstrate non-significant differences between the two experiments (*P* > 0.05). Data from the two experimental repetitions were pooled and significant differences among treatments were assessed with the Student’s t-test (*P* ≤ 0.05) and the Tukey’ test (*P* ≤ 0.05) in case of pairwise and multiple comparisons, respectively. CFU values of bacterial resolution were Log_10_-transformed and fold change values of gene expression analysis were Log_2_-transformed. The Pearson’s correlation coefficient between gene expression levels assessed by RNA-Seq and qPCR analysis was calculated with the Excel program.

## Results

### Endophytic Bacterial Strains and Humic Acid Enhance Tomato Growth

**Figure 1:**
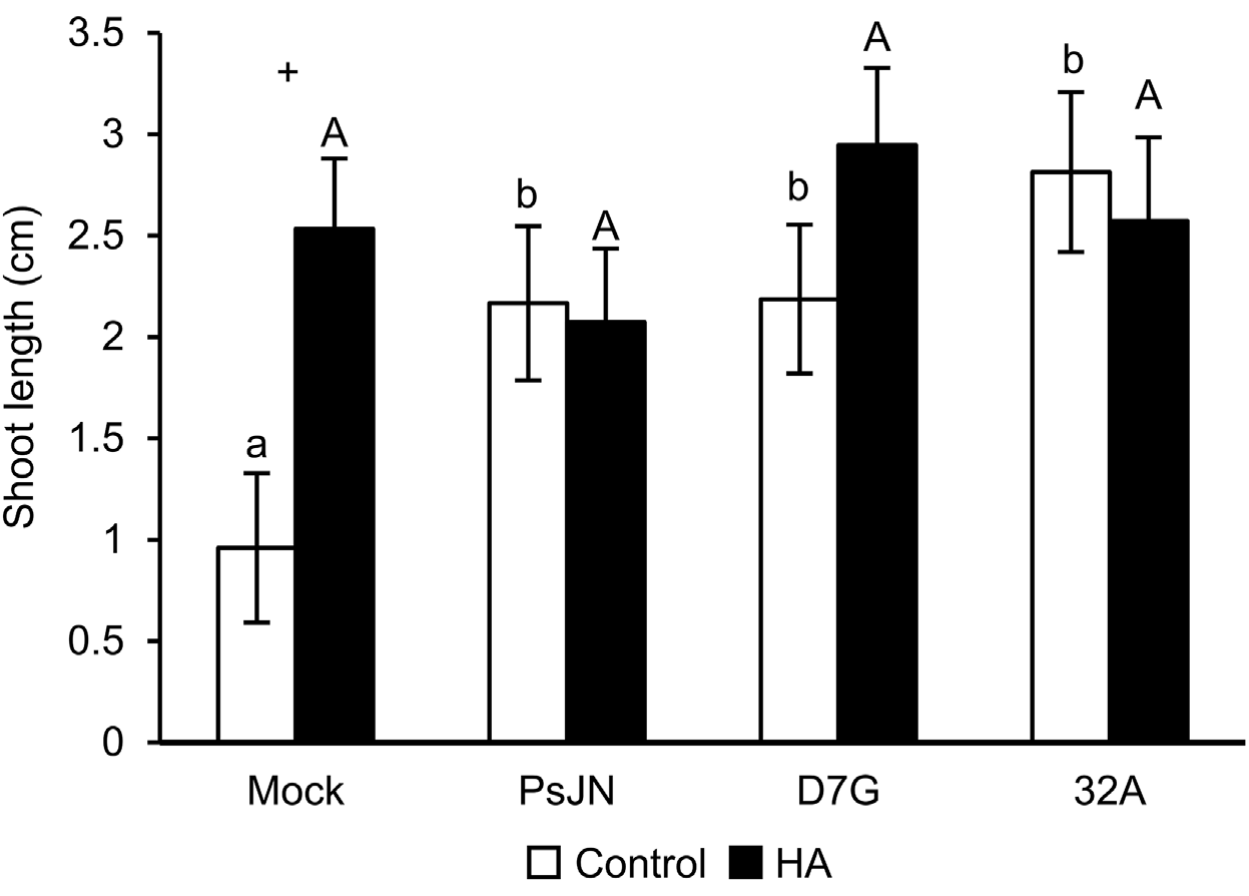
Tomato growth promotion by endophytic bacterial strains. The shoot length (cm) of mock-inoculated plants (mock) and plants inoculated with *Paraburkholderia phytofirmans* PsJN (PsJN), *Pantoea agglomerans* D7G (D7G), or *Enterobacter* sp. 32A (32A) was assessed 3 days after incubation in half-strength Hoagland with 0 mg L^−1^ (white, control) and 50 mg L^−1^ humic acid (black, HA) in square dishes. Mean and standard error values of nine replicates (plants) are presented for each treatment. Different lowercase and uppercase letters indicate significant differences among treatments in the control and HA condition according to Tukey’s test (*P* ≤ 0.05), respectively. For each treatment, plus symbols indicate significant differences in the pairwise comparisons between the control and HA condition according to Student’s t test (*P* ≤ 0.05).

HA improved tomato growth (**Figure S1**), the maximum growth promotion of shoot and root length was obtained with 50 mg L^−1^ HA and this dosage was selected as optimized HA concentration for the subsequent experiments (HA condition). All endophytic bacterial strains grew in presence of 50 mg L^−1^ HA (**Figure S2** and **Table S1**). The tomato shoot length was longer in PsJN-, D7G-, 32A-, and 11E-inoculated plants compared to mock-inoculated plants in the absence of HA (control condition; **Figure S3A**). Likewise, PsJN, D7G, and 32A improved tomato shoot length in the HA condition and these three strains were selected for the subsequent experiments. Plants were colonized by the endophytic bacterial strains tested and PsJN, D7G, and 32A were re-isolated from surface-sterilized tomato plants at 6 DAI at comparable levels in the control and HA condition (**Figure S3B**). Tomato shoot length was promoted by PsJN, D7G, and 32A at 3 DAI in the control condition and it was also stimulated by HA in mock-inoculated plants, through a possible complementation of the endophytic PGPB and the organic biostimulant (**Figure 1**). Moreover, PsJN, D7G, and 32A confirmed the promotion of tomato shoot length at 6 DAI in the HA condition and indicated different effects of growth promotion according to the incubation time (**Figure S4**).

**Figure 2:**
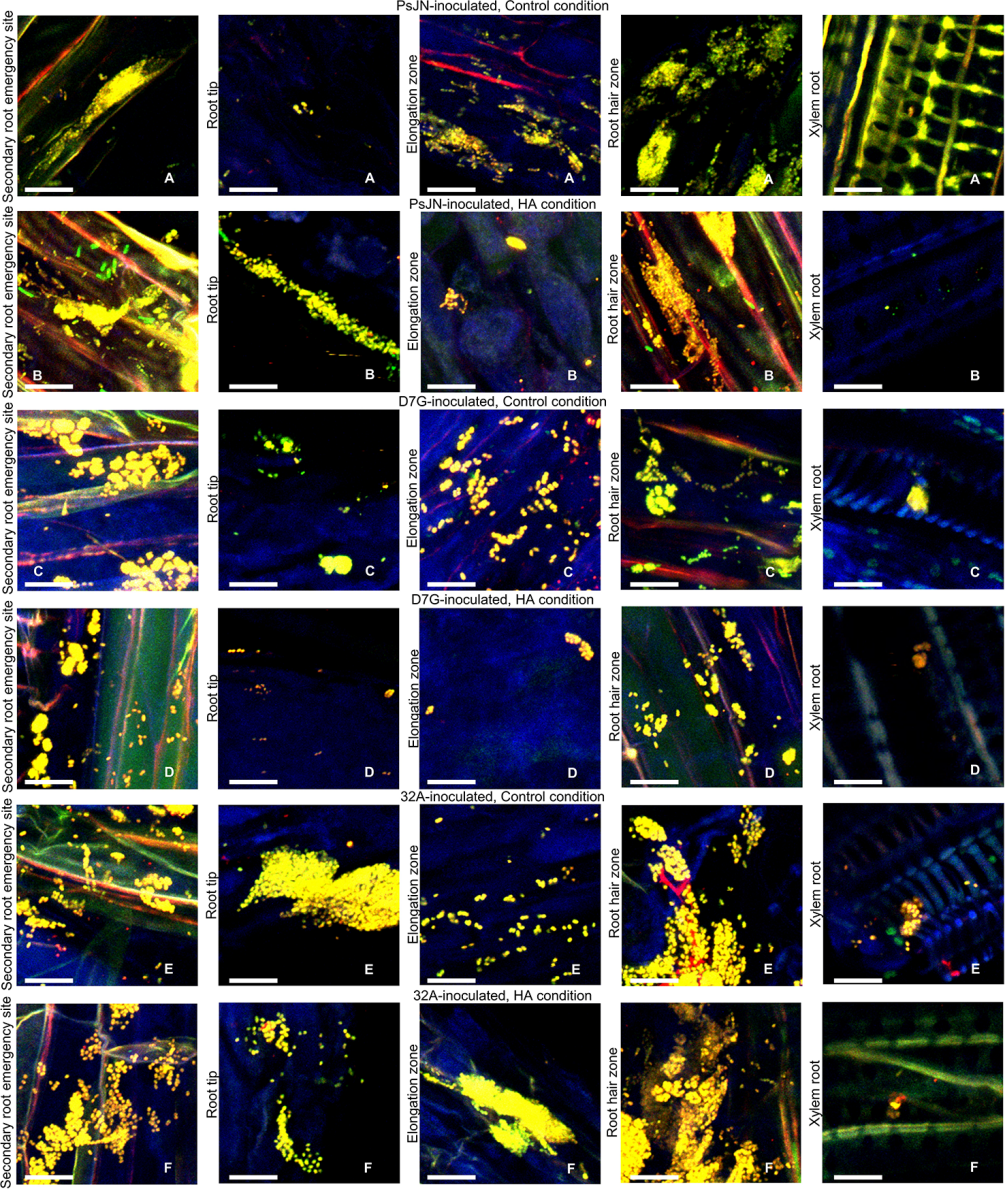
Location of endophytic bacterial strains on and inside tomato roots. Bacterial cells of *Paraburkholderia phytofirmans* PsJN (PsJN) **(A, B)** were hybridized with the EUBmix and Bphyt probes, *Pantoea agglomerans* D7G (D7G) **(C, D)**, or *Enterobacter* sp. 32A (32A) **(E, F)** were hybridized with the EUBmix and Gam42a probes on secondary root emergency sites (a), root tip zone (b), root elongation zone (c), root hair zone (d), and xylem (e) 3 days after incubation (DAI) in half-strength Hoagland with 0 mg L^−1^ (Control condition; **A**, **C**, **E**) and 50 mg L^−1^ humic acid (HA condition; **B**, **D**, **F**) in square dishes. Five replicates (plants) were analyzed for each treatment and representative pictures were selected. Bars correspond to 10 µM.

To better characterize the colonization of tomato tissues by endophytic bacterial strains, the DOPE-FISH analysis was carried out in the control and HA condition using specific probes targeting the 23S rRNA gene and universal probes for bacteria. Yellow fluorescent PsJN (**Figures 2A, B**), D7G (**Figures 2C, D**) and 32A (**Figures 2E, F**) single cells, aggregates, and micro-colonies were found on the secondary root emergency site, root tip, root elongation zone, root hair, and xylem of tomato roots in the control and HA condition. PsJN, D7G, and 32A cells were also found on the tomato stem and xylem in the control and HA condition (**Figure S5**) and the colonization intensity of tomato roots among the tested strains were comparable in the control and HA condition at 3 DAI (**Figure 2**) and 6 DAI (**Figure S6**). In mock-inoculated plants only some native bacteria were present (**Figure S7**). The NONEUB probe was used as negative probe not targeting bacterial sequences and only a few green/blue-cyan/orange/reddish autofluorescent microbes could be seen in mock-, PsJN-, D7G-, and 32A-inoculated plants as indication of the rare presence of native autofluorescent microorganisms (**Figures S7, S8**).

### Endophytic Bacterial Strains and Humic Acid Modulate Tomato Genes in Roots and Shoots

**Figure 3:**
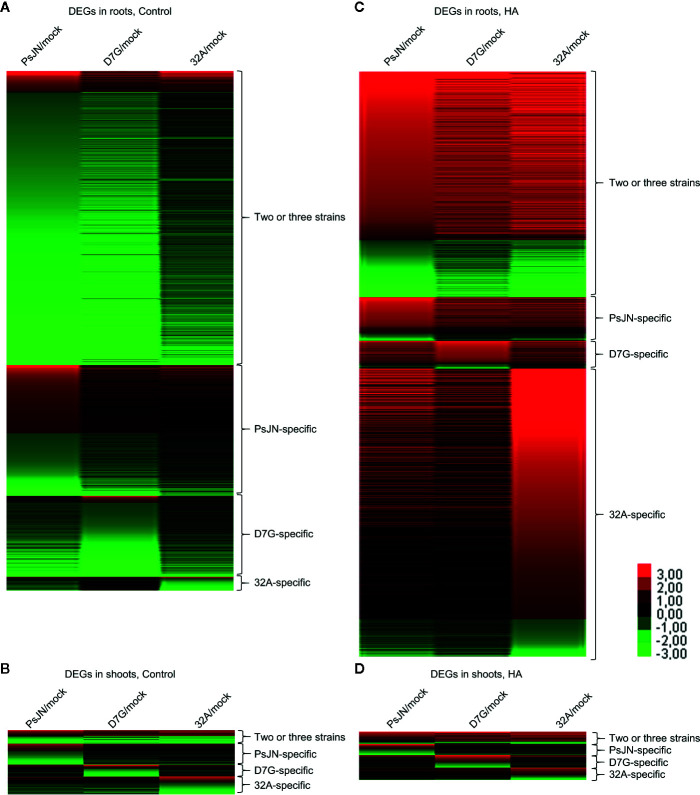
Clustering of differentially expressed genes (DEGs) of tomato plants in response to endophytic bacterial strains and humic acid. Heat map diagram indicates the fold change values for DEGs identified in tomato roots **(A, C)** and shoots **(B, D)** 3 days after incubation with *Paraburkholderia phytofirmans* PsJN (PsJN), *Pantoea agglomerans* D7G (D7G), or *Enterobacter* sp. 32A (32A), calculated as compared to mock-inoculated plants (mock) in half-strength Hoagland with 0 mg L^−1^ (control; **A**, **C**) and 50 mg L^−1^ humic acid (HA; **B**, **D**). DEGs were classified as genes modulated by two or three strains or as genes modulated by only one bacterial strain (PsJN-, D7G-, or 32A-specific). The heat map diagram was visualized using Java Treeview according to color scale legend shown.

To further characterize the plant response to endophytic bacterial strains and HA, a transcriptomic analysis of tomato shoots and roots was carried out. From 11.7 to 23.8 million reads were obtained for each replicate of tomato shoots and roots collected from mock-inoculated plants and plants inoculated with PsJN, D7G, and 32A at 3 DAI in the control and HA condition (**Table S3**). More than 80.0% of tomato genes were expressed in at least one condition (**Table S4**). A total of 6,135 and 623 DEGs were identified in tomato roots and shoots respectively, according to the pairwise comparisons between bacterium-inoculated (PsJN-, D7G-, and 32A-inoculated) and mock-inoculated plants in the control and HA condition, while 4,227 and 422 genes were modulated by HA in mock-inoculated roots and shoots, with a *P*-value lower than 0.01 and minimum Log_2_-transformed fold change of one (**Tables S5–S10**). The majority of DEGs was downregulated (79.4%) by endophytic bacterial strains in the control condition. Conversely, DEGs were mainly upregulated (80.0%) by endophytic bacterial strains in the HA condition, as a consequence of a possible HA-dependent enhancement of tomato reactions to endophytic bacterial strains (**Figure 3**). DEGs were grouped in genes modulated by at least two endophytic bacterial strains (DEGs modulated by two or three strains), to highlight possible common reactions to bacterial endophytes, or specifically by only one endophytic bacterial strain (PsJN-, D7G-, or 32A-specific DEGs), to highlight possible strain-specific reactions, in roots or shoots in the control and HA condition (**Figures 3**, **S9**). The RNA-Seq results were validated by a qPCR analysis of 10 tomato genes (**Table S2**) that were selected according to their expression profiles [five genes modulated in roots and five in shoots; five modulated only in the control condition and three modulated only in the HA condition and belonging to one of the four different clusters (modulated by two or three strains, PsJN-specific, D7G-specific or 32A-specific)] and functional categories (e.g., defense, growth and development, hormone metabolism, oxidative stress, protein metabolism, secondary metabolism, transcription, and transport). A close correlation (Pearson correlation coefficient, 0.93) between RNA-Seq and qPCR expression data was observed (**Figure S10**). In particular, expression profiles generated by qPCR and RNA-Seq agreed completely for eight genes and differed slightly for two genes (**Table S2**), possibly due to differences in the method sensitivity and discrimination capacity of multigene families (Perazzolli et al., 2016).

### Endophytic Bacterial Strains Activate a Complex Transcriptional Response in Tomato Roots According to the Presence of Humic Acid

In tomato roots, 539 and 3,688 genes were upregulated and downregulated by HA in mock-inoculated plants, respectively (**Figure S11A and Table S5**). A significant enrichment of GO categories related to regulation of metabolic process and regulation of transcription was found for genes upregulated by HA (**Figure S11D**), such as transcription factors (e.g., 12 MYB, seven WRKY, five NAC domain-containing and two ethylene-responsive transcription factors) and signal transduction-related genes (e.g., 14 kinases, eight calcium-binding proteins, and four receptor kinases; **Figure S11C** and **Table S5**). Moreover, genes downregulated by HA in tomato roots indicated global repression of cellular metabolic processes and energy-related processes (**Figure S11E**).

**Figure 4:**
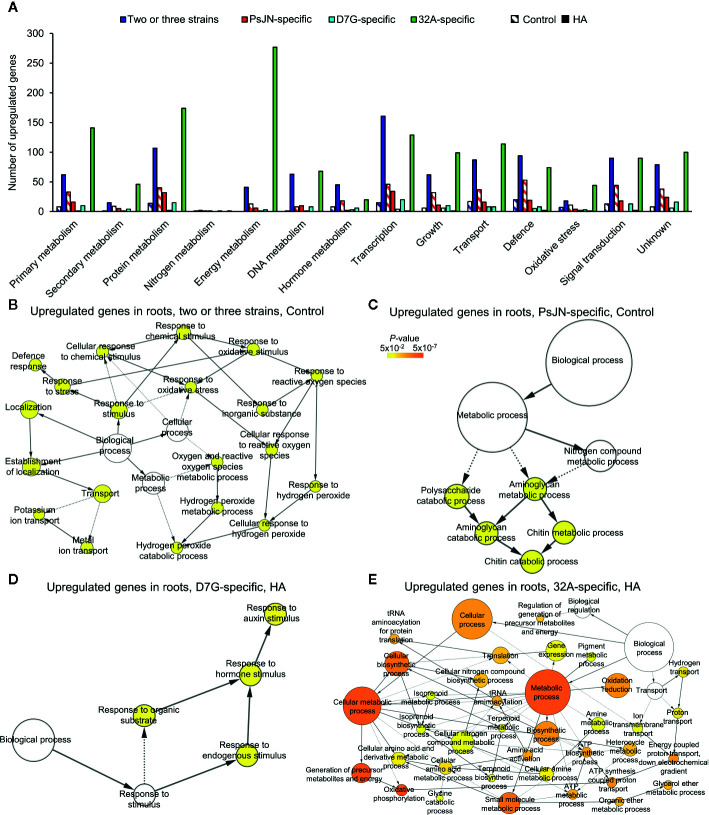
Functional annotation of upregulated genes in tomato roots in response to endophytic bacterial strains. Functional classes **(A)** were assigned on the basis of the protein description of upregulated genes in tomato roots in response to two or three strains (blue) and specifically in response to *Paraburkholderia phytofirmans* PsJN (red), *Pantoea agglomerans* D7G (cyan), or *Enterobacter* sp. 32A (green) in half-strength Hoagland with 0 mg L^−1^ (control; stripped bars) and 50 mg L^−1^ humic acid (HA; solid bars). Biological networks of significantly enriched (*P* ≤ 0.05) Gene Ontology (GO) terms of upregulated genes in tomato roots in response to two or three strains **(B)** or to PsJN **(C)** in the control condition and in response to D7G **(D)** or 32A **(E)** in the HA condition are reported. The color scale legend indicates the level of significance for enriched GO terms and white nodes indicate not significantly overrepresented categories. Dotted lines indicate connection between biological process categories in the GO chart, where ancestor and child are omitted for simplicity. No significant GO enrichment was found for upregulated genes in response to two or three strains and to PsJN in the HA condition, as well as in response to D7G or 32A in the control condition.

There were 119 and 926 genes upregulated by two or three strains in the control and HA condition, respectively (**Tables S6, S7**). Genes upregulated by two or three strains in tomato roots in the control condition were mainly involved in protein metabolism (e.g., one cysteine desulfurase, three F-box proteins and one tyrosine aminotransferase), transcription [e.g., two basic helix-loop-helix transcription factors, (bHLH), three zinc finger proteins and two WRKYs], transport (e.g., one heavy metal transport protein, one iron-regulated transporter, three potassium channels, one potassium transporter and two vacuolar iron transporters), signal transduction (e.g., four kinases, one receptor kinase and two serine/threonine-protein kinases), and defense [e.g., four defensin-like proteins, two nucleotide-binding site leucine-rich repeat proteins (NBS-LRRs) and three leucine rich repeat (LRR) receptorlike proteins; **Figure 4A** and **Table S6**]. As a possible common reaction to bacterial endophytes, a significant enrichment of GO categories related to defense response, response to stimulus and oxidative stress (e.g., five peroxidases and one glutaredoxin) was found for upregulated genes by two or three strains in the control condition (**Figure 4B** **and** **Table S6**). The presence of HA enhanced the transcriptional changes activated in response to two or three strains, in terms of number of genes and FC values. Thus, genes related to protein metabolism, transcription [e.g., one WRKY, one ethylene (ET) response factor and two ET responsive transcription factors], transport, signal transduction (e.g., six receptor kinases, 17 protein kinases, three calcium transporting ATPases, one calcium-dependent protein kinase, one calcium/calmodulin-dependent serine/threonine-kinase, and 16 serine/threonine-protein kinases) and defense (e.g., four NBS-LRRs, four defensin-like proteins) were upregulated by two or three strains in the HA condition, together with genes implicated in the growth and development process (e.g., two cellulose synthases, six glycosyltransferases, one mannosyltransferase, two pectin lyases and three pectinesterases), in the hormone metabolism (e.g., four 1-aminocyclopropane-1-carboxylate synthases, three cytokinin riboside phosphoribohydrolases, one gibberellin oxidase, one gibberellin dioxygenase, one auxin efflux facilitator, and eleven small auxin responsive proteins), and response to oxidative stress (e.g., seven peroxidases, four glutaredoxins and one glutathione S-transferase; **Figure 4A** **and** **Table S7**).

**Figure 5:**
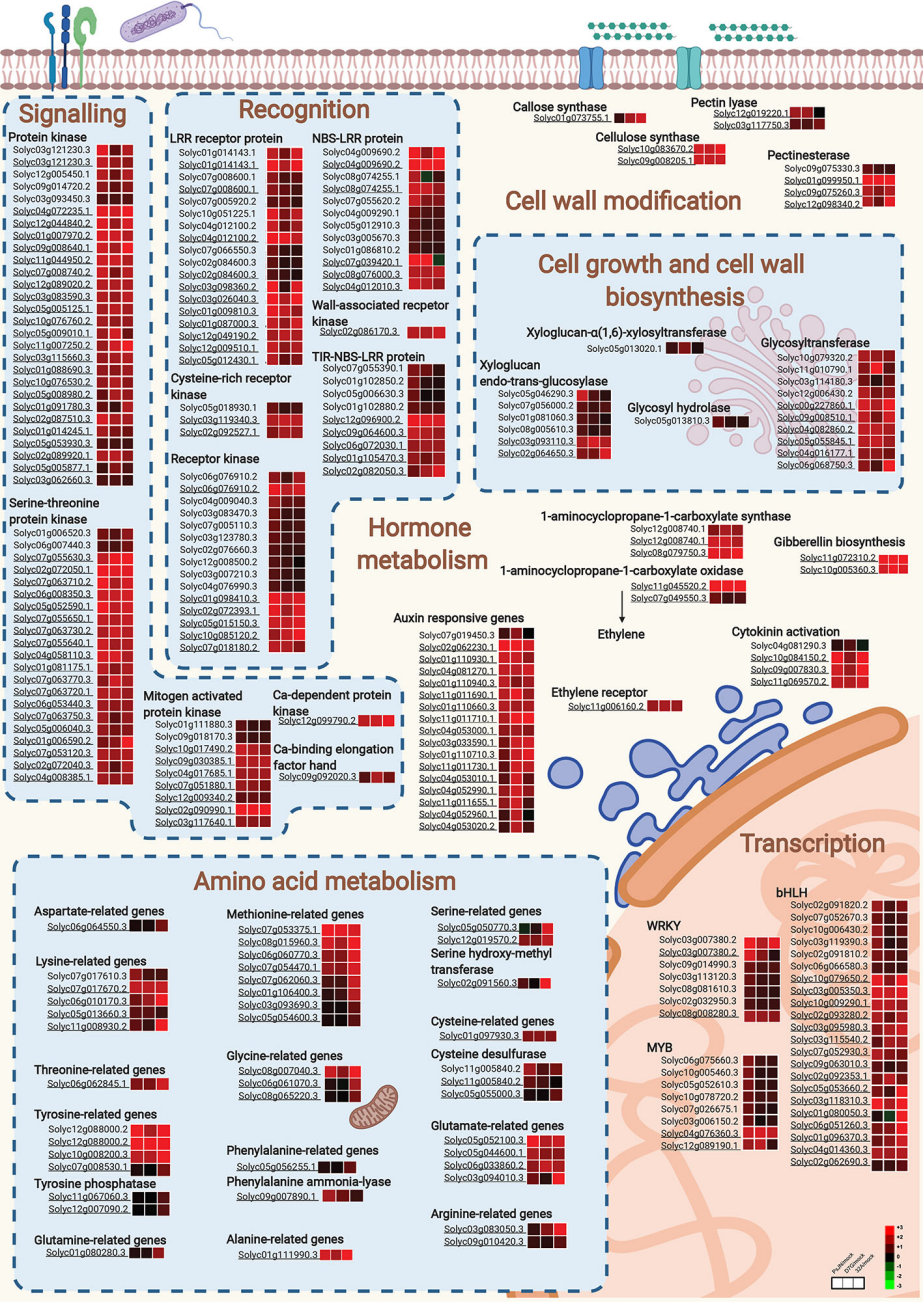
Cellular processes activated by endophytic bacterial strains in tomato roots. Main cellular pathways of upregulated genes in tomato roots in response to *Paraburkholderia phytofirmans* PsJN (PsJN), *Pantoea agglomerans* D7G (D7G) or *Enterobacter* sp. 32A (32A) in half-strength Hoagland with 0 mg L^−1^ (control) and 50 mg L^−1^ humic acid (HA) were generated with Biorender. Not underlined and underlined gene codes indicate tomato genes modulated in the control and HA condition, respectively. For each gene, three squares represent the Log_2_-transformed fold change values of PsJN-, D7G-, or 32A-inoculated plants calculated as compared to mock-inoculated plants respectively, according to the color scale reported. bHLH, basic helix-loop-helix; LRR, leucine-rich repeat; NBS-LRR, nucleotide-binding site leucine-rich repeat; TIR-NBS-LRR, non-toll-interleukin receptor nucleotide-binding site leucine-rich repeat; Ca, calcium.

PsJN-specific genes revealed the upregulation of genes related to protein metabolism (e.g., one lysine-ketoglutarate reductase and one threonine aldolase), transcription [e.g., four basic helix loop helix transcription factors (bHLHs), two basic-leucine zipper family proteins (bZIP), six MYBs and three WRKY transcription factors and five zinc finger proteins], transport (e.g., two mannose transporter, one phosphate transporter and one potassium transporter), defense (e.g., two disease resistance proteins, four LRR receptor like proteins and six NBS-LRRs), signal transduction (e.g., nine kinases and eight receptor kinases), and hormone metabolism in the control condition (**Figure 4A**). As a consequence, the GO categories related to the chitin metabolic process and the aminoglycan and polysaccharide catabolic processes were enriched in the cluster of PsJN-specific genes in the control condition (**Figure 4C**). In addition, PsJN-specific genes upregulated in the HA condition were involved in protein metabolism (e.g., one cysteine desulfurase, one glutamate dehydrogenase, and 10 F-box proteins), transcription (e.g., two bHLHs, five zinc finger proteins, one MYB and two WRKYs), defense (e.g., one disease resistance proteins, three LRR receptor like proteins and one phenylalanine ammonia-lyase), and signal transduction (e.g., four serine/threonine-kinases, one histidine kinase, four protein kinases and one receptor kinase), as possible enhancement of tomato response in the HA condition (**Figure 4A**). D7G-specific genes upregulated in the control condition were involved in transport, defense, growth, and development (**Figure 4A**), while those upregulated in the HA condition were mainly involved in protein metabolism (e.g., one cysteine synthase and one glutamate dehydrogenase), transcription (e.g., one bHLH and two MYB transcription factors), and signal transduction (e.g., three protein kinases). In particular, D7G-specific genes involved in the response to hormone stimulus were upregulated in the HA condition (e.g., five small auxin responsive proteins and an ET receptor; **Figure 4D**) and control condition (e.g., 1-aminocyclopropane-1-carboxylate synthase, auxin-regulated IAA protein, cytokinin hydrolase). Likewise, 32A-specific genes upregulated in the HA condition were mainly involved in protein metabolism, energy metabolism (e.g., nine NADH dehydrogenases, one cytochrome c oxidase, and one ATPase) and transcription (e.g., three ankyrin repeat family proteins, seven bHLHs, eight zinc finger proteins; **Figure 4A**). In particular, the GO categories related to secondary metabolism and amino acid metabolism were enriched (**Figure 4E**) in the cluster of 32A-specific genes in the HA condition, together with genes related to oxidative stress response (e.g., 14 thioredoxins, three glutathione S-transferases, three superoxide dismutases, three glutaredoxins, and two peroxidases; **Figure 4A** **and** **Table S7**). Thus, the cellular processes involved in tomato root response to endophytic bacterial strains in the control and HA condition revealed the activation of a complex recognition machinery that involves signal transduction pathways and the consequent activation of transcription-, protein-, transport-, and defense-related pathways (**Figures 5**, **S12**). Different recognition processes were activated by PsJN, D7G, and 32A, and the presence of HA enhanced the upregulation of signal transduction, hormone metabolism, transcription, protein metabolism, transport, defense, and growth-related process in terms of number of DEGs and fold change values. Conversely, genes downregulated by endophytic bacterial strains in tomato roots suggest fine regulation of protein metabolism, DNA metabolism and secondary metabolism in the control and HA condition (**Figure S13**).

### Endophytic Bacterial Strains Activate a Complex Transcriptional Response in Tomato Shoots According to the Presence of Humic Acid

HA incubation caused the upregulation and downregulation of 52 and 170 genes in tomato shoots of mock-inoculated plants, respectively (**Figure S11B and Table S8**). Tomato genes upregulated by HA were involved in primary metabolism (**Figure S11C**) and indicated the activation of the GO categories related to carbohydrate metabolism, alcohol metabolism and cell wall macromolecule metabolism (**Figure S11F**). Conversely, genes related to ROS metabolism were mainly downregulated by HA (**Figure S11G**).

**Figure 6:**
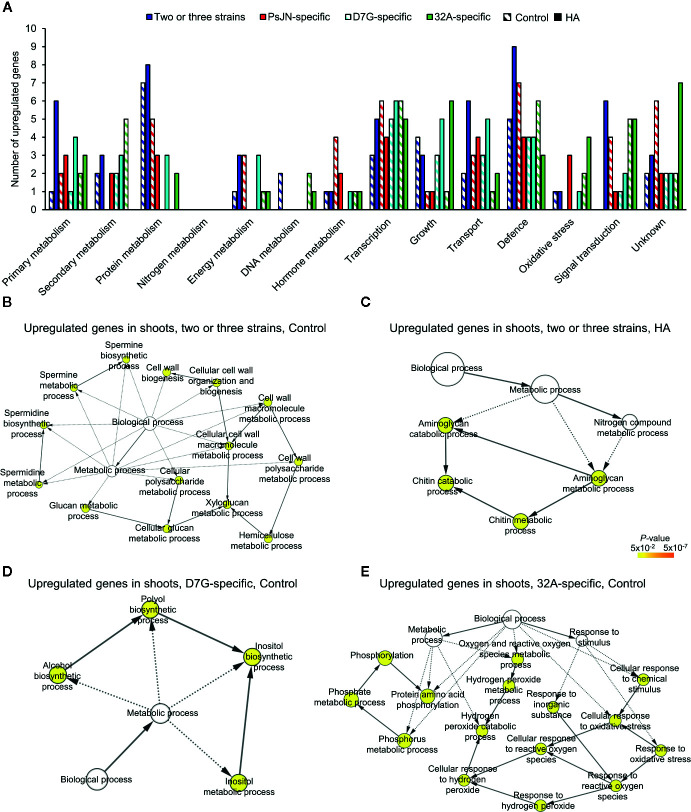
Functional annotation of upregulated genes in tomato shoots in response to endophytic bacterial strains. Functional classes **(A)** were assigned on the basis of the protein description of upregulated genes in tomato shoots in response to two or three strains (blue) and specifically in response to *Paraburkholderia phytofirmans* PsJN (red), *Pantoea agglomerans* D7G (cyan), or *Enterobacter* sp. 32A (green) in half-strength Hoagland with 0 mg L^−1^ (control; stripped bars) and 50 mg L^−1^ humic acid (HA; solid bars). Biological networks of significantly enriched (*P* ≤ 0.05) Gene Ontology (GO) terms of upregulated genes in tomato shoots in response to two or three strains in the control condition **(B)** and HA condition **(C)** and in response to D7G **(D)** or 32A **(E)** in the control condition are reported. The color scale legend indicates the level of significance for enriched GO terms and white nodes indicate not significantly overrepresented categories. Dotted lines indicate connection between biological process categories in the GO chart, where ancestor and child are omitted for simplicity. No significant GO enrichment was found for upregulated genes in response to PsJN in the control and HA condition, as well as in response to D7G or 32A in the HA condition.

Genes upregulated by two or three strains in tomato shoots in the control condition were mainly related to protein metabolism, defense, growth and development (**Figure 6A** **;** **Tables S9, S10**). A significant enrichment of the GO categories related to cell growth was found for upregulated genes by two or three strains in the control condition, such as cell wall-related processes (e.g., one glucan synthase and two xyloglucan endotransglucosylases; **Figure 6B**). In the HA condition, the enrichment of the GO categories related to aminoglycan metabolism and chitin metabolism was found for upregulated genes by two or three strains (**Figure 6C**). Genes associated to primary metabolism (e.g., two 2-oxoglutarate oxygenases and one lipase), protein metabolism (e.g., one calreticulin and one cysteine desulfurase), and transport (e.g., one calcium transporting ATPase) were upregulated by two or three strains in the HA condition (**Figure 6A**), in agreement with the shoot length promotion.

**Figure 7:**
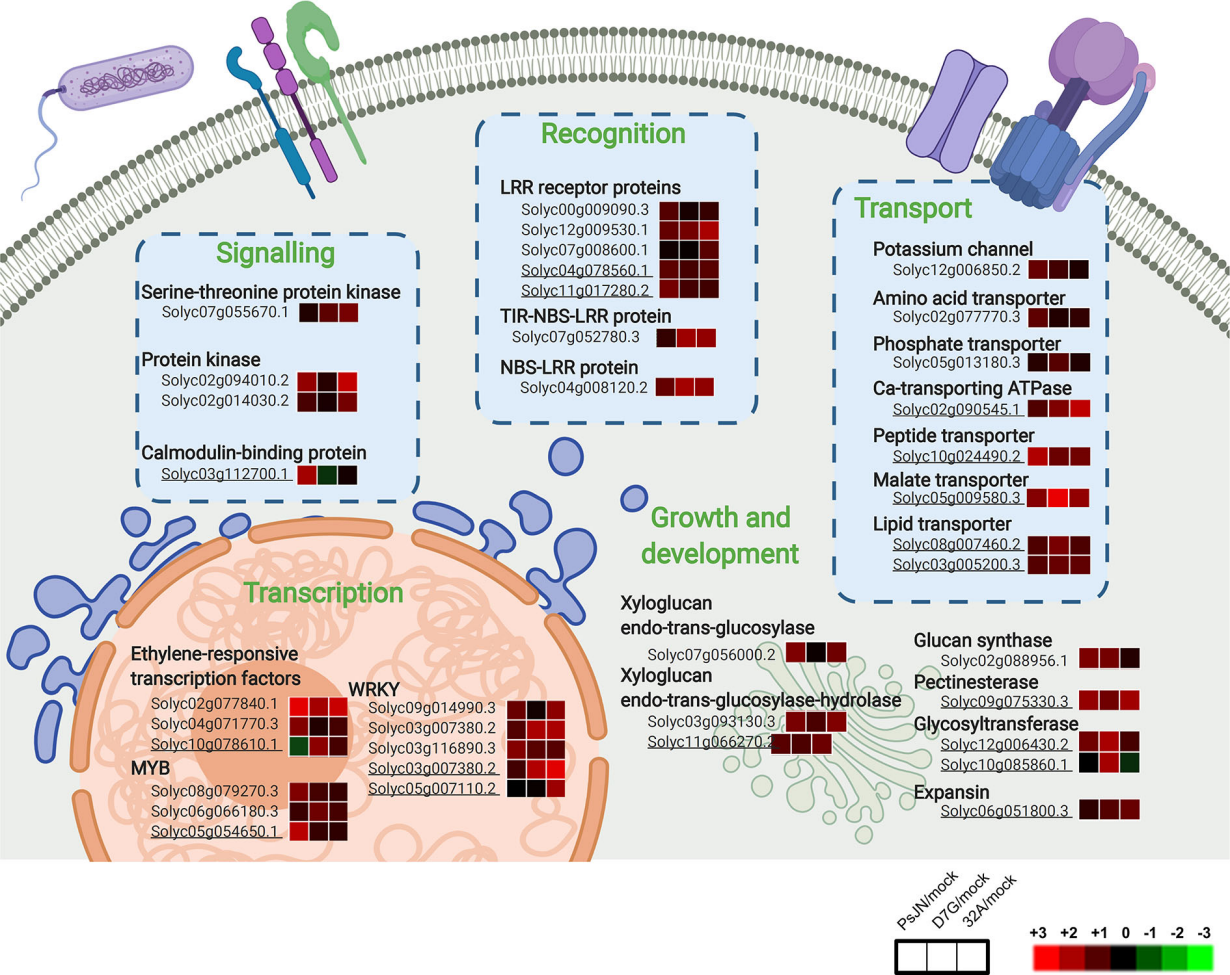
Cellular processes activated by endophytic bacterial strains in tomato shoots. Main cellular pathways of upregulated genes in tomato shoots in response to *Paraburkholderia phytofirmans* PsJN (PsJN), *Pantoea agglomerans* D7G (D7G), or *Enterobacter* sp. 32A (32A) in half-strength Hoagland with 0 mg L^−1^ (control) and 50 mg L^−1^ humic acid (HA) were generated with Biorender. Not underlined and underlined gene codes indicate tomato genes modulated in the control and HA condition, respectively. For each gene, three squares represent the Log_2_-transformed fold change values of PsJN-, D7G-, or 32A-inoculated plants calculated as compared to mock-inoculated plants respectively, according to the color scale reported. Ca, calcium; LRR, leucine-rich repeat receptor proteins; NBS-LRR, nucleotide-binding site leucine-rich repeat proteins; TIR-NBS-LRR, non-toll-interleukin receptor nucleotide-binding site leucine-rich repeat proteins; StkP, serine-threonine protein kinase.

PsJN-specific genes modulated in tomato shoots were mainly related to protein metabolism, transcription and defense in the control condition (**Figure 6A**). Similarly, PsJN-specific genes upregulated in the HA condition were involved in transcription (e.g., one MYB and one zinc finger protein), defense, and transport. Tomato processes related to transcription, growth and development (e.g., one cyclin and one cell division cycle protein), transport, and defense were also upregulated by D7G in the control condition (**Figure 6A**), with the enrichment of the inositol and polyol GO processes (**Figure 6D**). In the HA condition, genes involved in transcription (e.g., one ET responsive transcription factor), growth and development (e.g., one expansin and two glycosyltransferases), and transport (e.g., one aluminium-activated malate transporter and two lipid transfer proteins) were upregulated by D7G. Moreover, 32A-specific genes upregulated in the control condition were mainly involved in the secondary metabolism and defense (**Figure 6A**) and the GO categories related to oxidative stress and phosphorylation were enriched (**Figure 6E**). 32A-specific genes related to transcription (e.g., one WRKY transcription factor), growth and development (e.g., one expansin, one xyloglucan hydrolase and one xyloglucan endoglucanase inhibitor), signal transduction (e.g., two LRR kinases), and oxidative stress response (e.g., one glutathione S-transferase and one peroxidase) were upregulated in the HA condition (**Figure 6A** and **Table S10**). In summary, cellular processes activated in tomato shoots in response to endophytic bacterial strains included recognition-, signal transduction-, and transcription-related pathways, with an enhancement of transport- and growth-related processes in the HA condition (**Figure 7**). On the other hand, downregulated genes in tomato shoots were related to stress response and DNA metabolism in the control condition, as well as lipid transport in the HA condition (**Figure S14**).

## Discussion

Some strains belonging to the bacterial genera *Enterobacter*, *Pantoea*, and *Paraburkholderia* had already been previously recognized as PGPB (Sessitsch et al., 2005; Campisano et al., 2014; Hardoim et al., 2015) and this study demonstrated that seed inoculation with PsJN, D7G, and 32A promotes tomato shoot growth. Inoculated tomato plants were efficiently colonized by the tested endophytic bacterial strains and HA did not increase the tissue colonization compared to the control condition. Moreover, the addition of HA (at the optimal concentration of 50 mg L^−1^) enhanced the tomato growth induced by the endophytic bacterial strains, suggesting some possible complementation effects of HA to the tested PGPB. HA was known to improve nutrient uptake in tomato plants (Adani et al., 1998; Dursun et al., 2002), by increasing electrolyte leakage, cell permeability and nutrient accumulation (David et al., 1994) and activating primary and secondary metabolism (Aguiar et al., 2018; Canellas et al., 2019). HA incubation upregulated genes responsible for cellular regulations in mock-inoculated plants, such as transcription factors, receptors and kinases, and altered the transcriptional response of tomato plants to endophytic bacterial strains.

Tomato genes were modulated by endophytic bacterial strains mainly in roots (2,919 and 3,216 in the control and HA condition, respectively) compared to shoots (355 and 268 in the control and HA condition respectively), indicating major transcriptional regulations in belowground compared to aboveground tissues. The majority of DEGs was downregulated (79.4%) by endophytic bacterial strains in the control condition. Conversely, DEGs were mainly upregulated (80.0%) by endophytic bacterial strains in the HA condition, suggesting enhanced reactions of tomato plants to bacterial endophytes in the presence of HA. In particular, the majority of genes upregulated by the endophytic bacterial strains in roots in the HA condition was not modulated (64.2%) or downregulated (33.1%) in roots in the control condition, while only 2.7% was upregulated, but with lower extent, also in the control condition, indicating that specific genes are implicated in the tomato response to bacterial endophytes in the presence of HA. In particular, the presence of HA enhanced the activation of signal transduction, hormone metabolism, transcription, protein metabolism, transport, defense, and growth-related processes in response to PsJN, D7G, and 32A inoculation, as better discussed in the following paragraphs. Moreover, half of the DEGs (45.5%) was modulated by at least two endophytic bacterial strains and they represent possible common pathways modulated in response to bacterial endophytes.

### Transcriptional Response of Tomato Roots and Shoots to Two or Three Endophytic Bacterial Strains and Humic Acid

Plant roots play a critical role in perception and recognition of the rhizosphere microorganisms (De Palma et al., 2019) and the presence of HA enhanced the activation of signal transduction and transcription processes in response to endophytic bacterial strains. These functional categories were activated by two or three strains and they included genes encoding receptor kinases, protein kinases and NBS-LRR proteins, indicating the activation of a common recognition machinery to bacterial endophytes in tomato roots. In particular, serine/threonine kinases were upregulated by HA and two or three strains in roots, and protein kinases were also involved in HA-induced signaling in rice (Ramos et al., 2015) and *A. thaliana* (Trevisan et al., 2011). The elevation of intracellular calcium is also an indicator of plant response to beneficial microorganisms (Vadassery and Oelmüller, 2009) and a modulation of calcium- and calmodulin-related genes was found in response to HA alone and two or three strains in the HA condition.

Since beneficial effects of endophytic bacterial strains can derive from multiple mode of action (Glick, 2012; Ferreira et al., 2019), it is difficult to discriminate effects of microbial activities in providing nutrients to plants and/or direct stimulation of plant growth (e.g., modulation of the hormone levels). The increase of nutrient uptake was known as one of the mechanisms of plant growth promotion caused by PGPB (Glick, 2012) and HA (Zanin et al., 2019). In this study, tomato genes related to potassium and iron transport were upregulated by two or three strains in the control condition and genes related to magnesium, nitrogen, phosphate, sulphate, and zinc transport were upregulated in the HA condition, which makes them possible markers of tomato biostimulation. We found that ATPase-encoding genes were upregulated by two or three strains in the HA condition and by HA alone, and membrane pumps were previously found as activated by humic substances in tomato (Zandonadi et al., 2016) and maize (Quaggiotti et al., 2004), suggesting a positive effect of HA on tomato nutrient uptake.

Another mechanism of PGPB-dependent plant growth promotion is the modulation of the hormone levels (Glick, 2012). Tomato genes related to jasmonic acid (JA) response (e.g., WRKY transcription factors and defensins) were upregulated by two or three strains in the control and HA condition, while those related to ET synthesis (e.g., 1-aminocyclopropane-1-caroxylate synthases) and ET response (ET response factor and ET responsive transcription factors) were mainly upregulated in the HA condition. The interplay of auxin and ET signaling pathways was found also in the PsJN-dependent *A. thaliana* growth promotion (Poupin et al., 2016) and some auxin-responsive genes (e.g., auxin efflux facilitator and auxin responsive proteins) were upregulated by two or three strains in the HA condition and by HA alone. Likewise, the WAT1-related genes were upregulated by endophytic bacterial strains in both conditions and these genes are known to be involved in auxin transport and homeostasis, as well as in growth promotion and cell wall development (Irizarry and White, 2018), indicating a complex hormonal response to endophytic bacterial strains in the presence of HA. In particular, some genes implicated in gibberellin biosynthesis (e.g., copalyl diphosphate synthase, gibberellin oxidase, and gibberellin dioxygenase) and cytokinin metabolism (e.g., cytokinin riboside phosphoribohydrolases) were upregulated by HA alone and by two or three strains in the HA condition. PsJN, D7G, and 32A were able to produce auxin (Poupin et al., 2013; Campisano et al., 2014) and PsJN was able to induce gibberellin synthesis in *A. thaliana* (Poupin et al., 2013). Likewise, humic substances upregulated auxin responsive genes in *A. thaliana* (Trevisan et al., 2010) and showed cytokinin-like (Pizzeghello et al., 2012) and gibberellin-like (Nardi et al., 2000) activity in maize plants, suggesting additive effects of endophytic bacterial strains and HA in the stimulation of growth-related hormone metabolism in tomato.

As a possible consequence of hormonal changes, genes upregulated by at least two endophytic bacterial strains in the HA condition were involved in cell growth and cell wall biosynthesis, such as cellulose synthases, glycosyltransferases, mannosyltransferases, pectin lyases, pectinesterases, glucan synthase, and two xyloglucan endotransglucosylases as key markers of tomato biostimulation. Pectin and cellulose are implicated in cell wall expansion and the upregulation of genes encoding cell wall modification enzymes has been observed in growth promotion processes activated by *Pseudomonas fluorescens* in *A. thaliana* (Wang et al., 2005) and *Bacillus amyloliquefaciens* in cotton (Irizarry and White, 2018). Thus, the upregulation of genes encoding cell wall-loosening enzymes may be a common plant response to PGPB, in order to facilitate endophytic colonization and plant growth promotion (Irizarry and White, 2018). Markers of an attempted defense reaction and oxidative stress response were also upregulated by two or three strains in the control and HA condition. In particular, the upregulation of glutaredoxins, glutathione S-transferases, and peroxidases indicate the activation of the antioxidant machinery, as possible HA-dependent modulation of plant reaction to bacterial endophytes.

### Transcriptional Response of Tomato Roots and Shoots Specifically Activated by *Paraburkholderia phytofirmans* PsJN, *Pantoea agglomerans* D7G, or *Enterobacter* sp. 32A

Different signaling pathways were activated by PsJN, D7G, or 32A, indicating a strain-specific response activated in tomato plants. Receptor kinases and transcription factors (e.g., bHLH, bZIP, MYB, and WRKY) were upregulated specifically by PsJN in roots in the control and HA condition. Similarly, PsJN induced the expression of receptor-like kinase genes in swtichgrass (Lara-Chavez et al., 2015) and bZIP, MYB, and WRKY transcription factors in *A. thaliana* (Timmermann et al., 2019) as possible key regulators of plant response to PsJN. Moreover, D7G- and 32A-specific genes upregulated in tomato roots and shoots included a distinctive signal transduction (e.g., protein kinases) and transcription (e.g., bHLH, MYB, WRKY, and zinc finger transcription factors) process responsible for plant reaction to endophytic bacterial strains.

The strain-specific response of tomato involved the hormone metabolism. For example, the upregulation of salicylic acid (SA) biosynthesis (phenylalanine ammonia lyase) and SA responsive (e.g., pathogenesis-related genes) genes was found in PsJN-inoculated roots, suggesting SA accumulation in the HA condition, as previously shown in PsJN-inoculated switchgrass (Lara-Chavez et al., 2015). SA, JA, and ET were implicated in PsJN-induced resistance (Timmermann et al., 2019) and the interplay of ET with the auxin signaling pathways was responsible for PsJN-dependent growth promotion in *A. thaliana* (Poupin et al., 2016). The auxin signaling- (indole-3-acetic acid inducible and dormancy associated/auxin-repressed) and transport-related (auxin efflux facilitator) genes were upregulated by PsJN in tomato roots in the control and HA condition, respectively. The hormone-related genes were upregulated also by D7G in the control and HA condition (e.g., auxin responsive genes, ET-related receptor and transcription factor, auxin and cytokinin metabolic genes) and this endophytic strain showed ACC-deaminase activity and auxin production activity *in vitro* (Campisano et al., 2014). As possible additive effect, the presence of HA can affect the auxin-related processes, as shown in *A. thaliana* (Canellas et al., 2010) and tomato (Canellas et al., 2011) plants, suggesting a complementation effect of endophytic bacterial strains and HA.

The protein metabolic pathways were activated by 32A in tomato roots in the HA condition, indicating the activation of nitrogen assimilation with upregulation of genes related to the metabolism of lysine, serine, glycine, cysteine, tyrosine, threonine, glutamine, alanine, arginine, and methionine. Likewise, the nitrogen and secondary metabolism was activated in *H. seropedicae* HRC54-inoculated tomato plants in the presence of HA (Olivares et al., 2015) and the increased concentration of amino acids and secondary metabolites was found in sugarcane plants inoculated with *H. seropedicae* HRC54 and *G. diazotrophicus* PAL 5 in the presence of HA (Aguiar et al., 2018; Canellas et al., 2019). In particular, 32A was able to fix atmospheric nitrogen *in vitro* (Campisano et al., 2014) and it caused the upregulation of a glutamine synthetase gene in tomato roots in the HA condition. Glutamine synthase encoding genes were also upregulated by endophytic diazotroph bacteria in sugarcane (Nogueira et al., 2005) and an increased amino acid content was found in sugarcane inoculated with the diazotroph *Pantoea* sp. 9C strain (Loiret et al., 2009), suggesting that the activation of the amino acid metabolism contributes to plant growth promotion. Amino acids are key precursors of secondary metabolites and genes related to secondary metabolism were induced by 32A in the HA condition. In accordance with these findings, a previous study had demonstrated that 32A affected the accumulation of secondary metabolites in grapevine plants as a possible mechanism for the successful host colonization (Lòpez-Fernàndez et al., 2015a). Likewise, the precise tuning of the plant defense by the endophytic bacterial strains could contribute to a successful host colonization. For example, the antioxidant machinery was activated in tomato roots mainly in the HA condition, indicating the activation of an attempted defense reaction against endophytic bacterial strains that is probably tuned by the endophytic bacterial strains to allow tissue colonization.

## Conclusions

Growth promotion effects and transcriptional responses activated by bacterial endophytes in tomato plants were affected by the presence of HA, indicating a complementation effect of PGPB and the organic biostimulant under controlled conditions. In particular, HA enhanced the activation of pathways responsible for signal transduction, hormone metabolism, transcription, protein metabolism, transport, defense, and cell growth in response to the endophytic bacterial strains. Major transcriptional regulations occurred in tomato roots and involved global reactions activated by endophytic bacterial strains, including protein metabolism, transcription, transport, signal transduction, and defense processes. The optimized HA dosage and an in-depth knowledge of tomato reaction to bacterial colonization derived by this study represent key information for the further development of combined formulations of endophytic bacterial strains and HA as a tailored diet for tomato biostimulation. In addition, genes identified in this work may be the source of important markers of tomato biostimulation that can be used to monitor the plant response to bacterial endophytes and HA under field conditions.

## Data Availability Statement

The original contributions presented in the study are publicly available. This data can be found here: NCBI, PRJNA622763.

## Author Contributions

NG carried out the functional experiments, performed DOPE-FISH experiments and microscopy analyses, and wrote the manuscript. SC performed DOPE-FISH experiments and microscopy analyses. MM analyzed the RNA-Seq data. CS helped in the functional experiments. AS and FW revised the manuscript. IP revised the manuscript and analyzed the data. GP conceived the study and revised the manuscript. MP conceived the study, supervised the experiments, analyzed the data, and wrote the manuscript. All authors contributed to the article and approved the submitted version.

## Funding

This project has received funding from the European Union’s Horizon 2020 Research and Innovation Program under the Marie Skłodowska-Curie grant agreement no. 722642 (project INTERFUTURE).

## Conflict of Interest

NG and FW are employed by Biobest NV.

The remaining authors declare that the research was conducted in the absence of any commercial or financial relationships that could be construed as a potential conflict of interest.
