# Supplementary material for: Humic Acid Enhances the Growth of Tomato Promoted by Endophytic Bacterial Strains Through the Activation of Hormone-, Growth-, and Transcription-Related Processes
Source: Front Plant Sci. 2020 Sep 16;11:582267. doi: 10.3389/fpls.2020.582267 (PMC7524882; doi:10.3389/fpls.2020.582267)
Supplement: Supplementary file 1 [file DataSheet_1.pdf]

Supplementary materials

Humic acid enhances the growth of tomato promoted by endophytic bacterial strains through the activation of hormone-, growth- and transcription-related processes

Nikoletta Galambos[1,2,3], Stéphane Compant[4], Marco Moretto[1], Carmela Sicher[1], Gerardo Puopolo[1,5], Felix Wäckers[3], Angela Sessitsch[4], Ilaria Pertot[1,5] and Michele Perazzolli[1,5,*]

[1] Research and Innovation Centre, Fondazione Edmund Mach, Via E. Mach 1, 38010 San Michele all'Adige, Italy
[2] Department of Civil, Environmental and Mechanical Engineering, University of Trento, via Mesiano 77, 38123 Trento, Italy
[3] Biobest NV, Isle Velden 18, 2260, Westerlo, Belgium
[4]AIT Austrian Institute of Technology, Center for Health and Bioresources, Konrad Lorenz Straße 24, 3430 Tulln, Austria
[5]Center Agriculture Food Environment (C3A), University of Trento, via E. Mach 1, 38010 San Michele all'Adige, Italy

[*]Correspondence:

Michele Perazzolli, michele.perazzolli@unitn.it

Running title: Tomato response to endophytes and humic acid

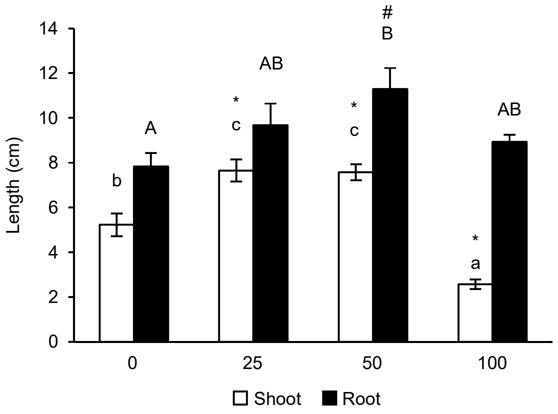

FIGURE S1 | Optimization of the humic acid concentration for tomato plants. The shoot (black) and root (white) length (cm) of tomato plants was assessed six weeks after incubation in half-strength Hoagland with 0 (control), 25, 50, 100 mg L^{-1} humic acid (HA) in glass tubes. The two-way analysis of variance showed no significant differences between the two experimental repetitions ($P > 0.05$, n = four replicates per experiment) and data from the two experiments were pooled. Mean and standard error values of eight replicates (plants) are presented for each treatment. Different lowercase and uppercase letters indicate significant differences in shoot and root length among treatments according to Tukey's test ($P \leq 0.05$), respectively. Asterisks and hashtags indicate significant differences in shoot and root length in the pairwise comparisons between the control and HA condition according to Student's t test ($P \leq 0.05$), respectively.

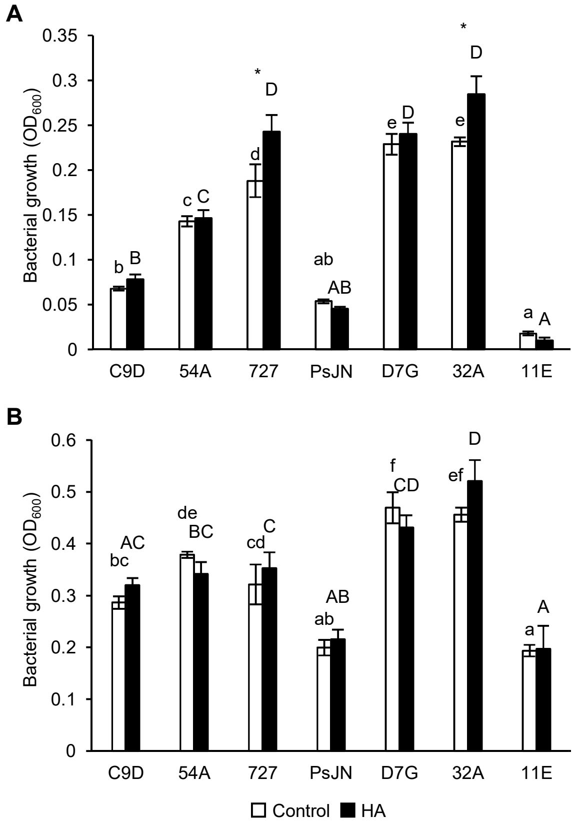

FIGURE S2 | Assessment of bacterial compatibility with humic acid. The bacterial growth (optical density at 600 nm, OD_{600}) of *Microbacterium* sp. C9D (C9D), *Bacillus* sp. 54A (54A), *Pantoea eucalypti* 727 (727), *Paraburkholderia phytofirmans* PsJN (PsJN), *Pantoea agglomerans* D7G (D7G), *Enterobacter* sp. 32A (32A) and *Sphingomonas* sp. 11E (11E) was assessed 12 h (A) and 24 h (B) after incubation in nutrient broth with 0 mg L^{-1} (white, control) and 50 mg L^{-1} humic acid (black, HA). The two-way analysis of variance showed no significant differences between the two experimental repetitions ($P > 0.05$, n = six replicates per experiment) and data from the two experiments were pooled. Mean and standard error values of 12 replicates (wells) are presented for each strain and treatment. Different lowercase and uppercase letters indicate significant differences among bacterial strains in the control and HA condition according to Tukey's test ($P \leq 0.05$), respectively. For each bacterial strain, asterisks indicate significant differences between the control and HA condition according to Student's t test ($P \leq 0.05$).

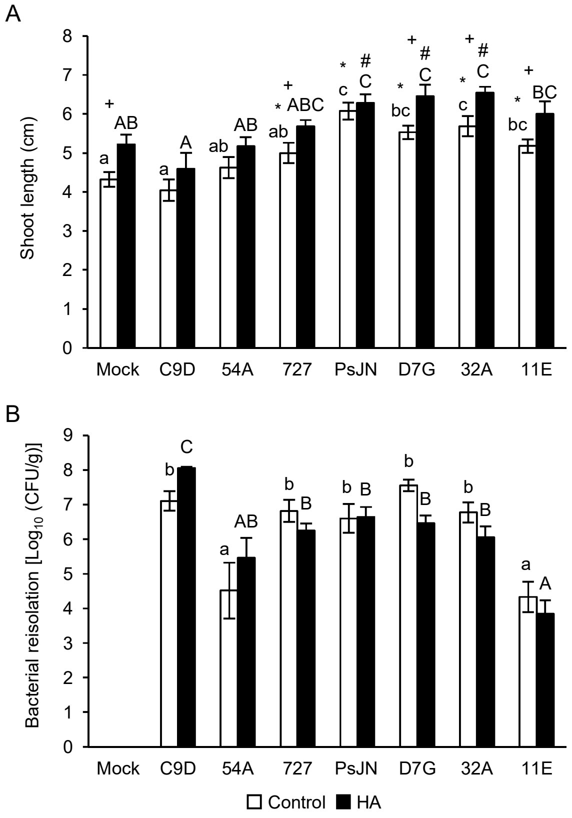

FIGURE S3 | Selection of the best tomato-growth promoting endophytic bacterial strains. The shoot length (cm) of tomato plants (A) and quantity of re-isolated bacterial strains (B), expressed as colony forming units per gram of fresh weight ($CFUg^{-1}$) were assessed for mock-inoculated plants (mock) and plants inoculated with *Microbacterium* sp. C9D (C9D), *Bacillus* sp. 54A (54A), *Pantoea eucalypti* 727 (727), *Paraburkholderia phytofirmans* PsJN (PsJN), *Pantoea agglomerans* D7G (D7G), *Enterobacter* sp. 32A (32A) or *Sphingomonas* sp. 11E (11E) six days after incubation in half-strength Hoagland with 0 mg L^{-1} (white, control) and 50 mg L^{-1} humic acid (black, HA) in square dishes. The two-way analysis of variance showed no significant differences between the two experimental repetitions ($P > 0.05$, n = five replicates per experiment) and data from the two experiments were pooled. Mean and standard error values of ten replicates (plants) are presented for each treatment. Different lowercase and uppercase letters indicate significant differences among treatments in the control and HA condition according to Tukey's test ($P \leq 0.05$), respectively. For each treatment, plus symbols indicate significant differences between the control and HA condition according to Student's t test ($P \leq 0.05$). Asterisks and hashtags of panel A indicate significant differences between bacterium-inoculated and mock-inoculated plants in the control and HA condition according to Student's t test ($P \leq 0.05$), respectively.

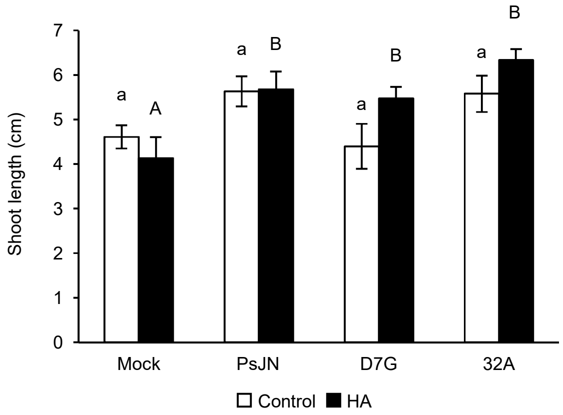

FIGURE S4 | Tomato growth promotion six days after incubation with endophytic bacterial strains. The shoot length (cm) of mock-inoculated plants (mock) and plants inoculated with *Paraburkholderia phytofirmans* PsJN (PsJN), *Pantoea agglomerans* D7G (D7G) or *Enterobacter* sp. 32A (32A) was assessed six days after incubation in half-strength Hoagland with 0 mg L^{-1} (control; white) and 50 mg L^{-1} humic acid (HA; black) in square dishes. Mean and standard error values of nine replicates (plants) are presented for each treatment. Different lowercase and uppercase letters indicate significant differences among treatments in the control and HA condition according to Tukey's test ($P \leq 0.05$), respectively.

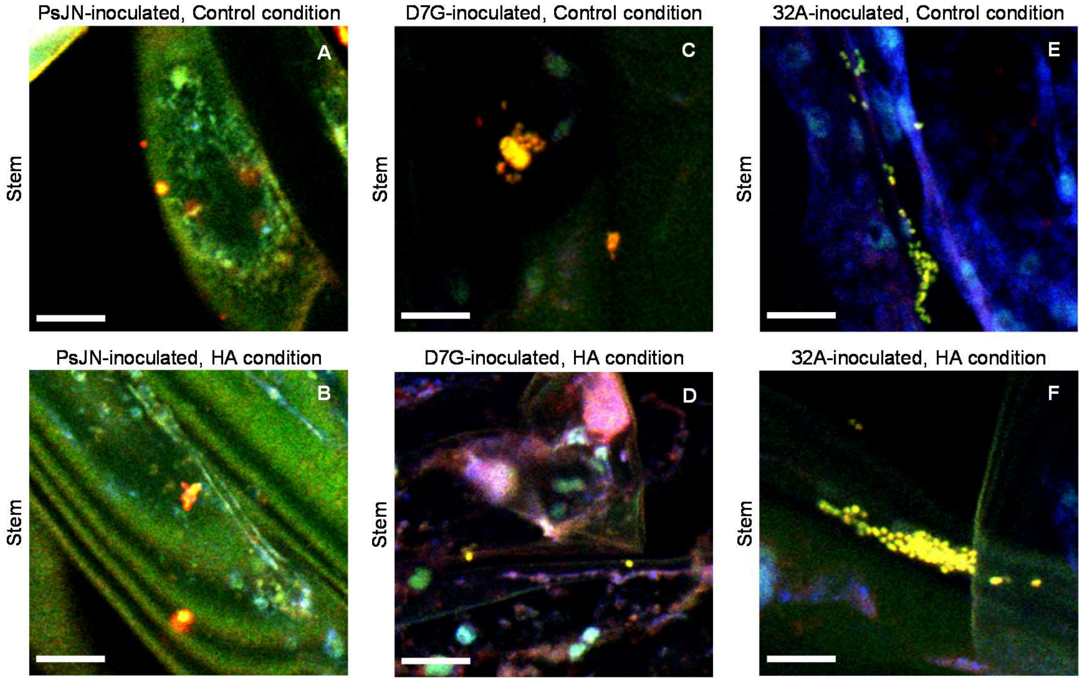

FIGURE S5 | Localization of endophytic bacterial strains on and inside tomato shoots. Bacterial cells of *Paraburkholderia phytofirmans* PsJN (PsJN) (A-B) were hybridized with the EUBmix and Bphyt probes, *Pantoea agglomerans* D7G (D7G) (C-D) or *Enterobacter* sp. 32A (32A) (E-F) were hybridized with the EUBmix and Gam42a probes on stem three days after incubation (DAI) in half-strength Hoagland with 0 mg L^{-1} (Control condition; A, C and E) and 50 mg L^{-1} humic acid (HA condition; B, D and F) in square dishes. Five replicates (plants) were analyzed for each treatment and representative pictures were selected. Bars correspond to 10 μM.

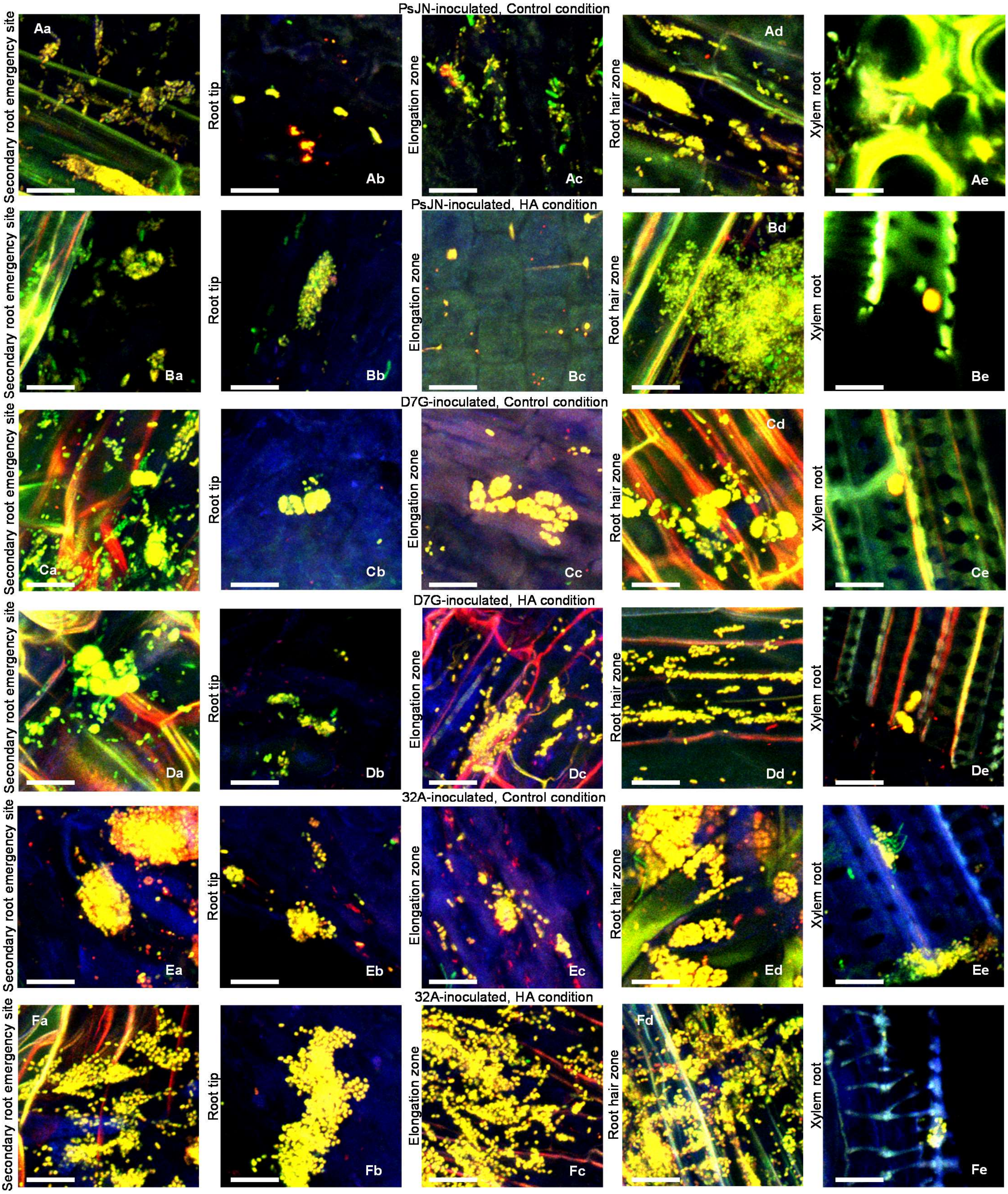

FIGURE S6 | Localization of endophytic bacterial strains on and inside tomato roots at six days after incubation. Bacterial cells of *Paraburkholderia phytofirmans* PsJN (PsJN) (A-B) were hybridized with the EUBmix and Bphyt probes, *Pantoea agglomerans* D7G (D7G) (C-D) or *Enterobacter* sp. 32A (32A) (E-F) were hybridized with the EUBmix and Gam42a probes on secondary root emergency sites (a), root tip (b), root elongation zone (c), root hair zone (d) and xylem (e) six days after incubation (DAI) in half-strength Hoagland with 0 mg L^{-1} (Control condition; A, C and E) and 50 mg L^{-1} humic acid (HA condition; B, D and F) in square

dishes. Five replicates (plants) were analyzed for each treatment and representative pictures were selected. Bars correspond to 10 μM.

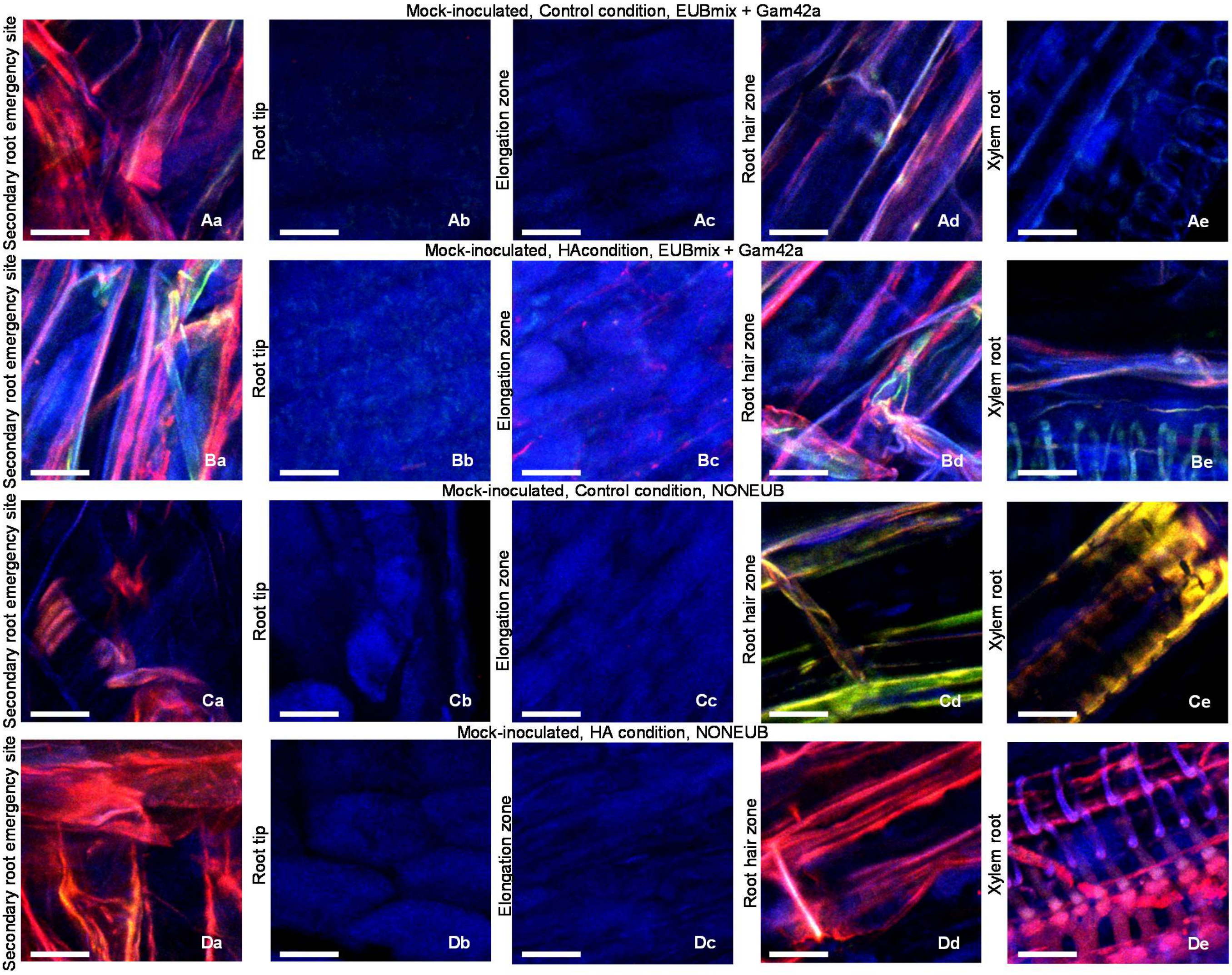

FIGURE S7 | Negative control of fluorescence in situ hybridization of mock-inoculated plants. Secondary root emergency sites (a), root tip (b), root elongation zone (c), root hair (d) and xylem (e) of mock-inoculated plants (mock) were hybridized with the EUBmix and Gam42a probe (A-B) and with the NONEUB probe (C-D) as negative probe not targeting bacterial sequences three days after incubation (DAI) in half-strength Hoagland with 0 mg L^{-1} (Control condition) and 50 mg L^{-1} humic acid (HA condition) in square dishes. Five replicates (plants) were analyzed for each treatment and representative pictures were selected. Bars correspond to 10 µM.

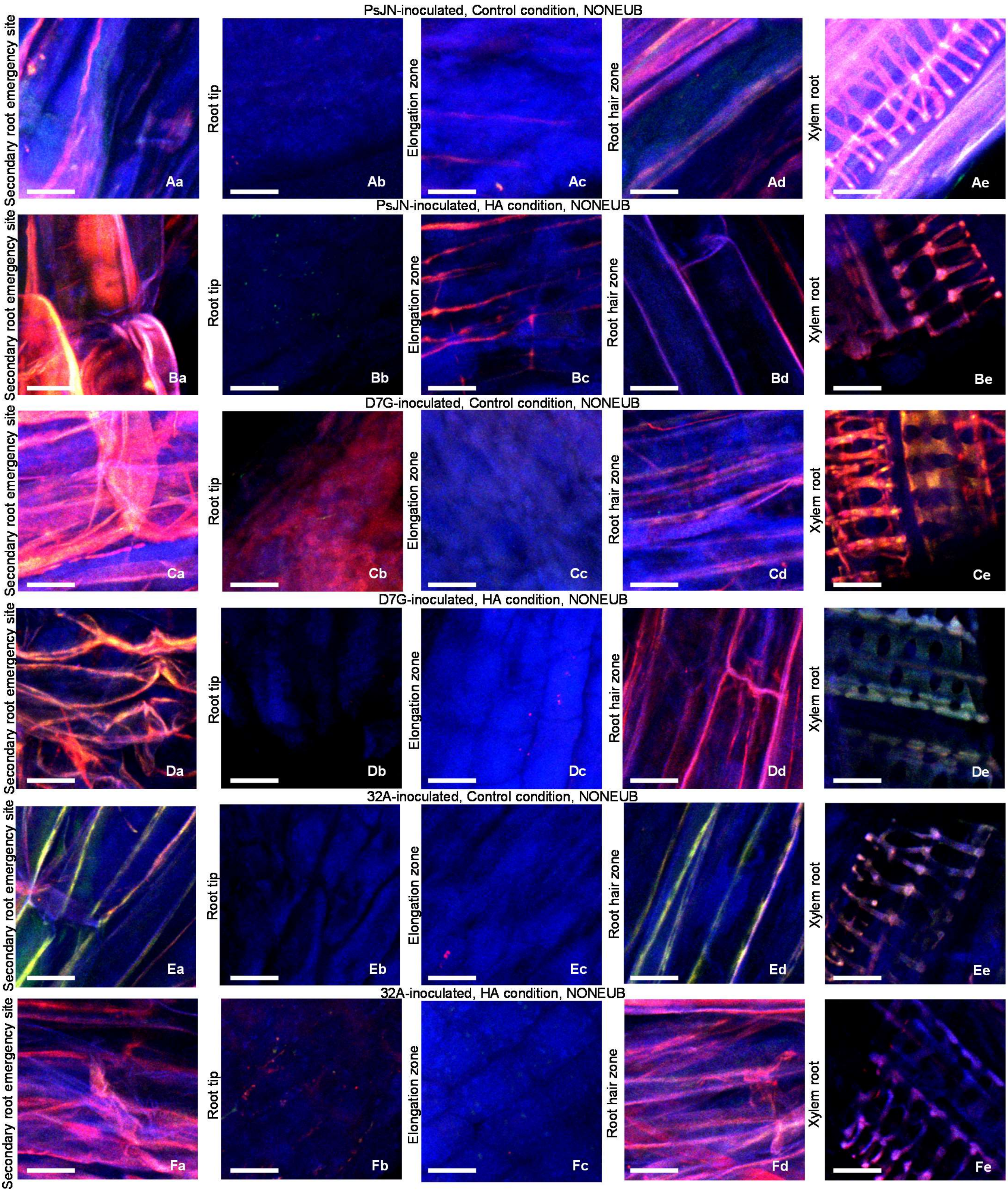

FIGURE S8 | Negative control of fluorescence in situ hybridization. Secondary root emergency sites (a), root tip (b), root elongation zone (c), root hair (d) and xylem (e) of plants inoculated with *Paraburkholderia phytofirmans* PsJN (PsJN; A-B), *Pantoea agglomerans* D7G (D7G; C-D) or *Enterobacter* sp. 32A (32A; E-F) were hybridized with the NONEUB probe as negative probe not targeting bacterial sequences, three days after incubation in half-strength Hoagland with 0 mg L^{-1} (Control condition) and 50 mg L^{-1} humic acid (HA condition) in square dishes. Five replicates (plants) were analyzed for each treatment and representative pictures were selected. Bars correspond to 10 µM.

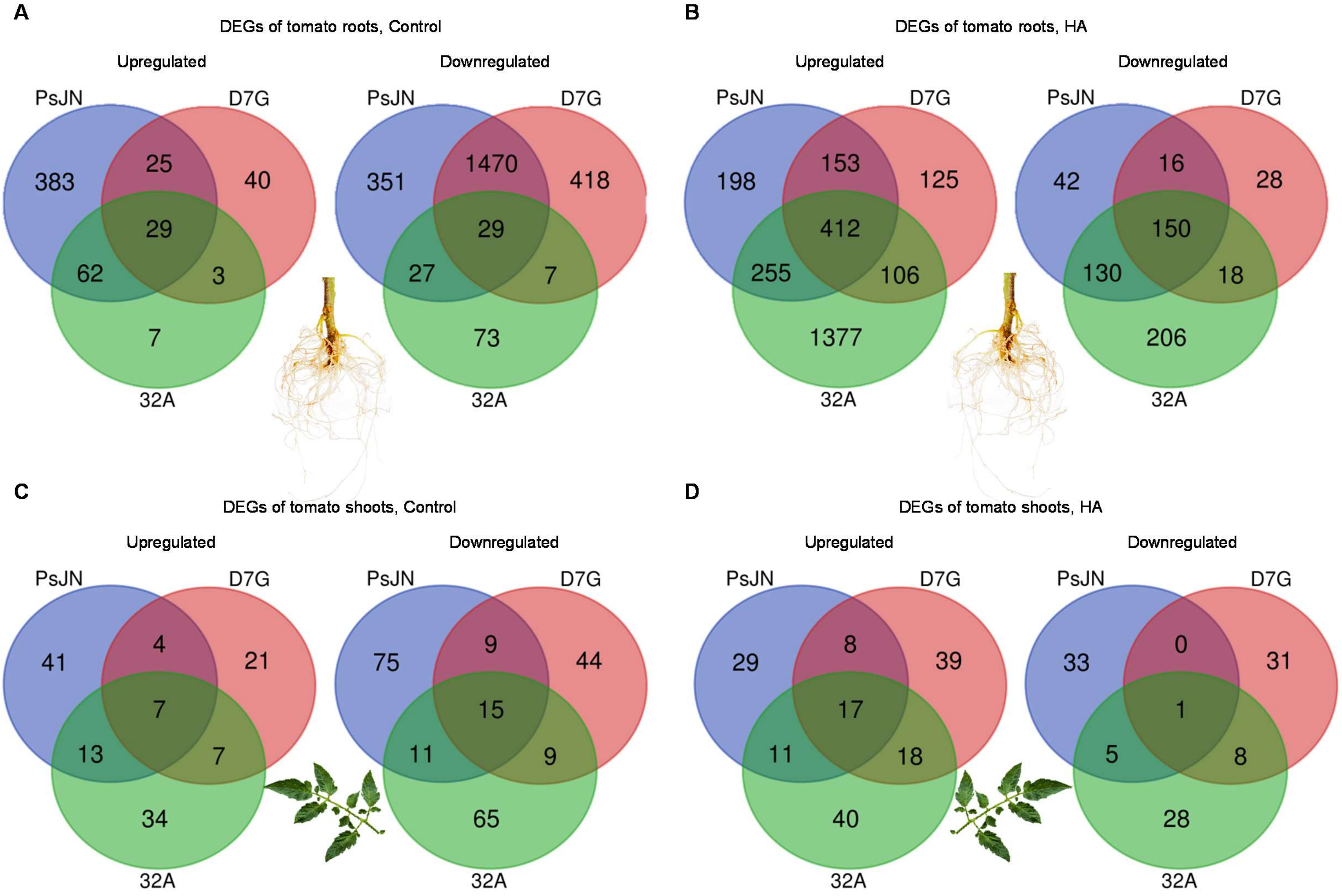

FIGURE S9 | Differentially expressed genes of tomato plants in response to endophytic bacterial strains and humic acid. Venn diagrams summarises the distribution of differentially expressed genes (DEGs) identified in tomato roots (A, B) and shoots (C, D) three days after incubation with *Paraburkholderia phytofirmans* PsJN (PsJN), *Pantoea agglomerans* D7G (D7G) or *Enterobacter* sp. 32A (32A) in comparison with the mock-inoculated plants in in half-strength Hoagland with 0 mg L^{-1} (control; A, C) and 50 mg L^{-1}humic acid (HA; B, D).

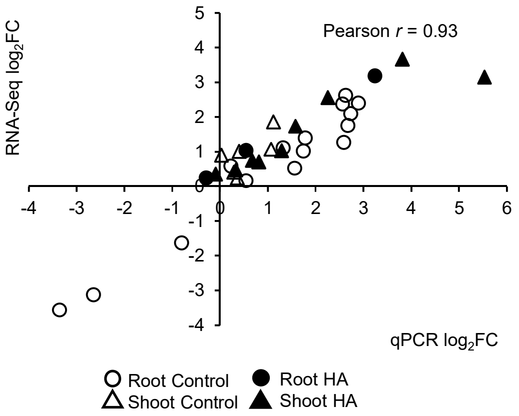

FIGURE S10 | Correlation analysis of gene expression data from RNA-Seq and quantitative real-time PCR. Scatter plot and Pearson correlation value (r) of Log_2-transformed fold change values assessed using the RNA-Seq and quantitative real-time PCR (qPCR) analysis of the selected genes (Table S2) in tomato roots (circle) and shoots (triangle) three days after incubation with *Paraburkholderia phytofirmans* PsJN, *Pantoea agglomerans* D7G or *Enterobacter* sp. 32A calculated as compared to mock-inoculated plants in half-strength Hoagland with 0 mg L^{-1} (control; white) and 50 mg L^{-1} humic acid (HA; black).

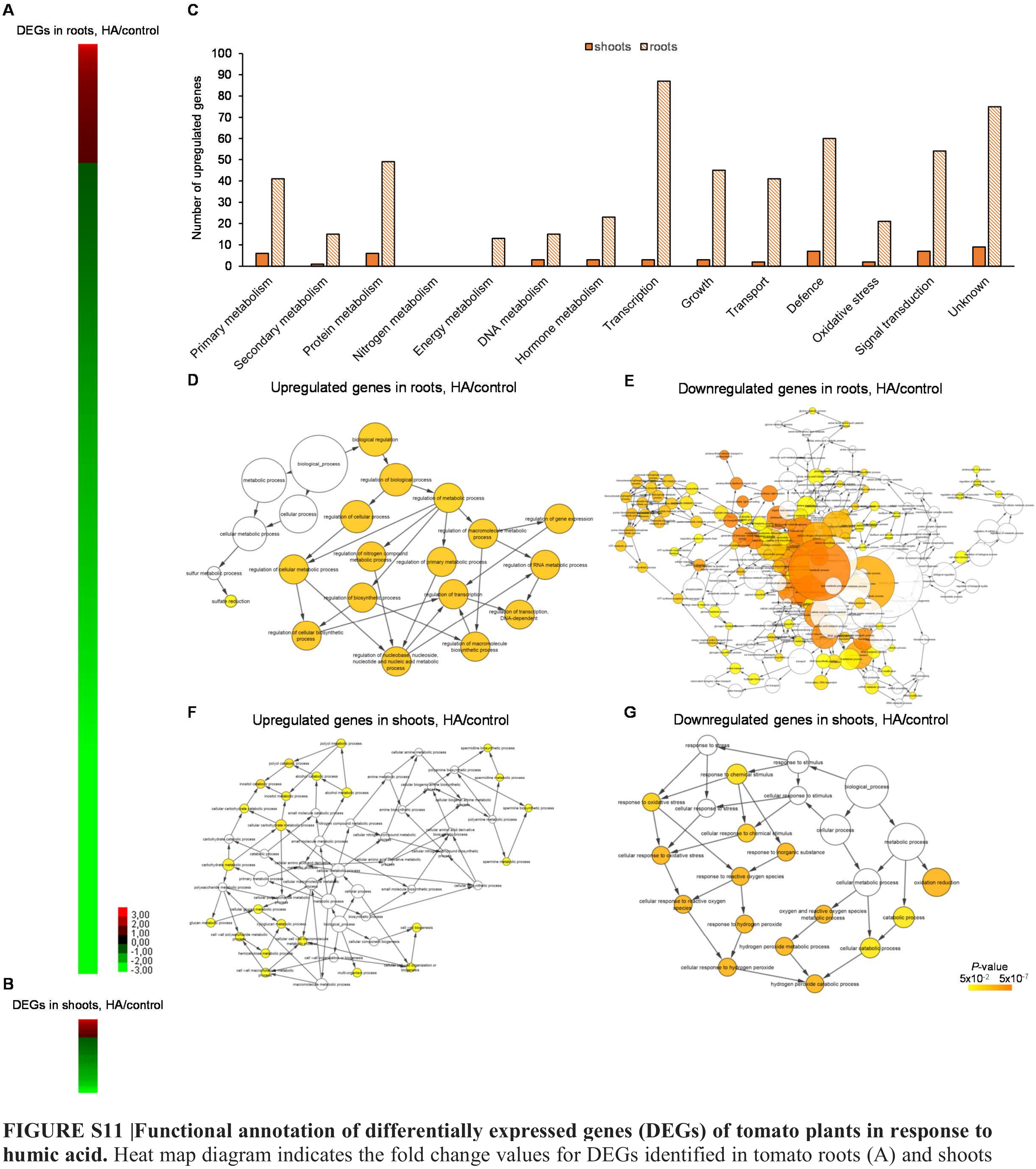

FIGURE S11 |Functional annotation of differentially expressed genes (DEGs) of tomato plants in response to humic acid. Heat map diagram indicates the fold change values for DEGs identified in tomato roots (A) and shoots (B) of mock-inoculated plants three days after incubation in half-strength Hoagland with 50 mg L^{-1} humic acid (HA) as compared to 0 mg L^{-1} HA (control). Functional classes (C) were assigned on the basis of the protein description of upregulated genes in tomato roots (stripped bars) and in shoots (solid bars) in response to HA. Biological networks of significantly enriched ($P \leq 0.05$) Gene Ontology (GO) terms of upregulated and downregulated genes in tomato roots (D, E) and shoots (F, G) in response to HA are reported. The colour scale legends indicate the fold changes and the level of significance for enriched GO terms. White nodes indicate not significantly overrepresented categories.

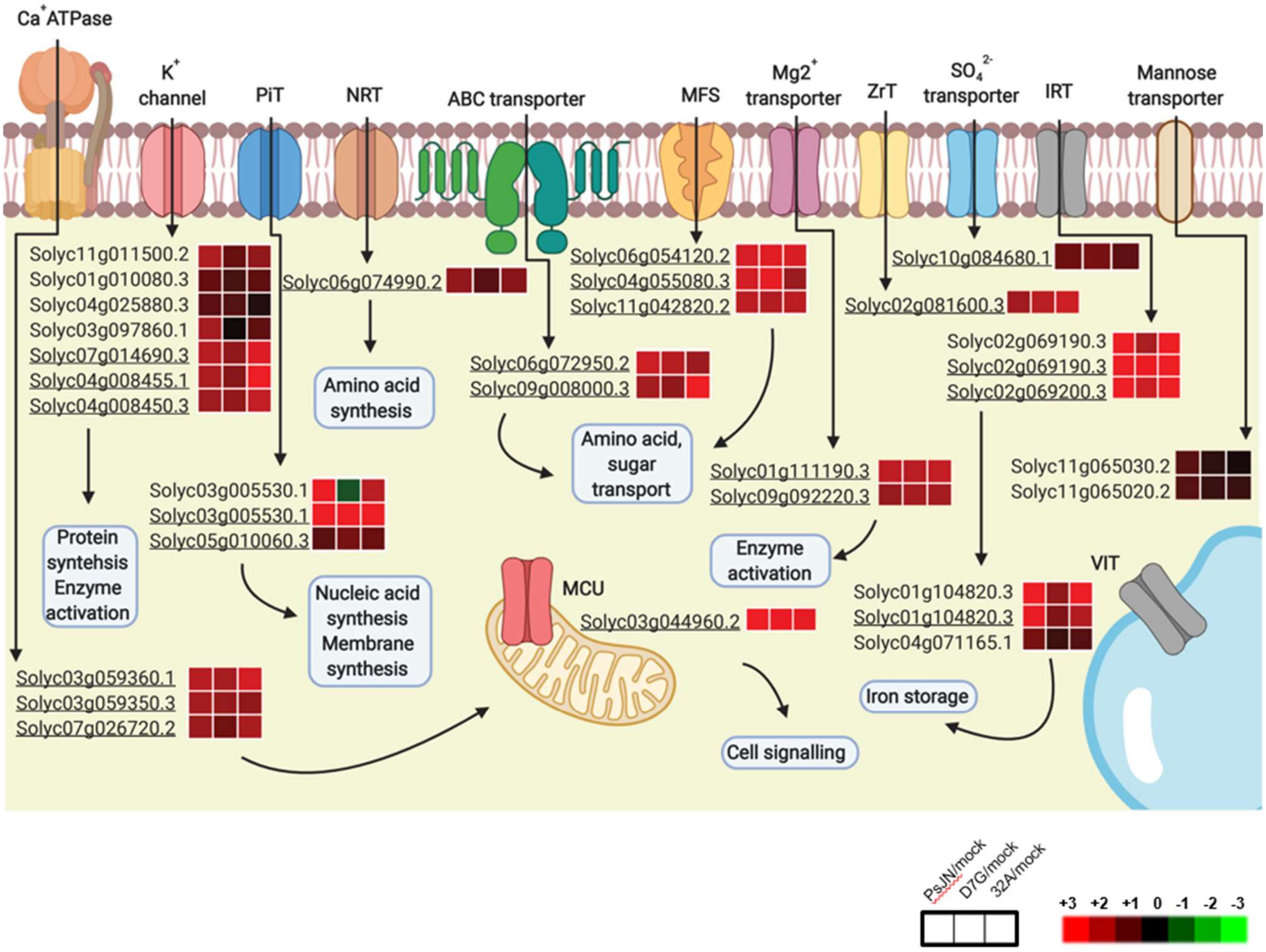

FIGURE S12 | Transport processes activated by endophytic bacterial strains in tomato roots. Transport processes of upregulated genes in tomato roots in response to *Paraburkholderia phytofirmans* PsJN (PsJN), *Pantoea agglomerans* D7G (D7G) or *Enterobacter* sp. 32A (32A) in half-strength Hoagland with 0 mg L^{-1} (control) and 50 mg L^{-1} humic acid (HA) were generated with Biorender. Not underlined and underlined gene codes indicate tomato genes modulated in the control and HA condition, respectively. For each gene, three squares represent the Log_2-transformed fold change values of PsJN-, D7G- or 32A-inoculated plants calculated as compared to mock-inoculated plants respectively, according to the colour scale reported. Abbreviations: PiT, phosphate transporter; NRT, high-affinity nitrate transporter; MFS, major facilitator superfamily protein; ZrT, zinc transporter protein; IRT, iron-regulated transporter; MCU, mitochondrial calcium uniporter protein; VIT, vacuolar iron transporter.

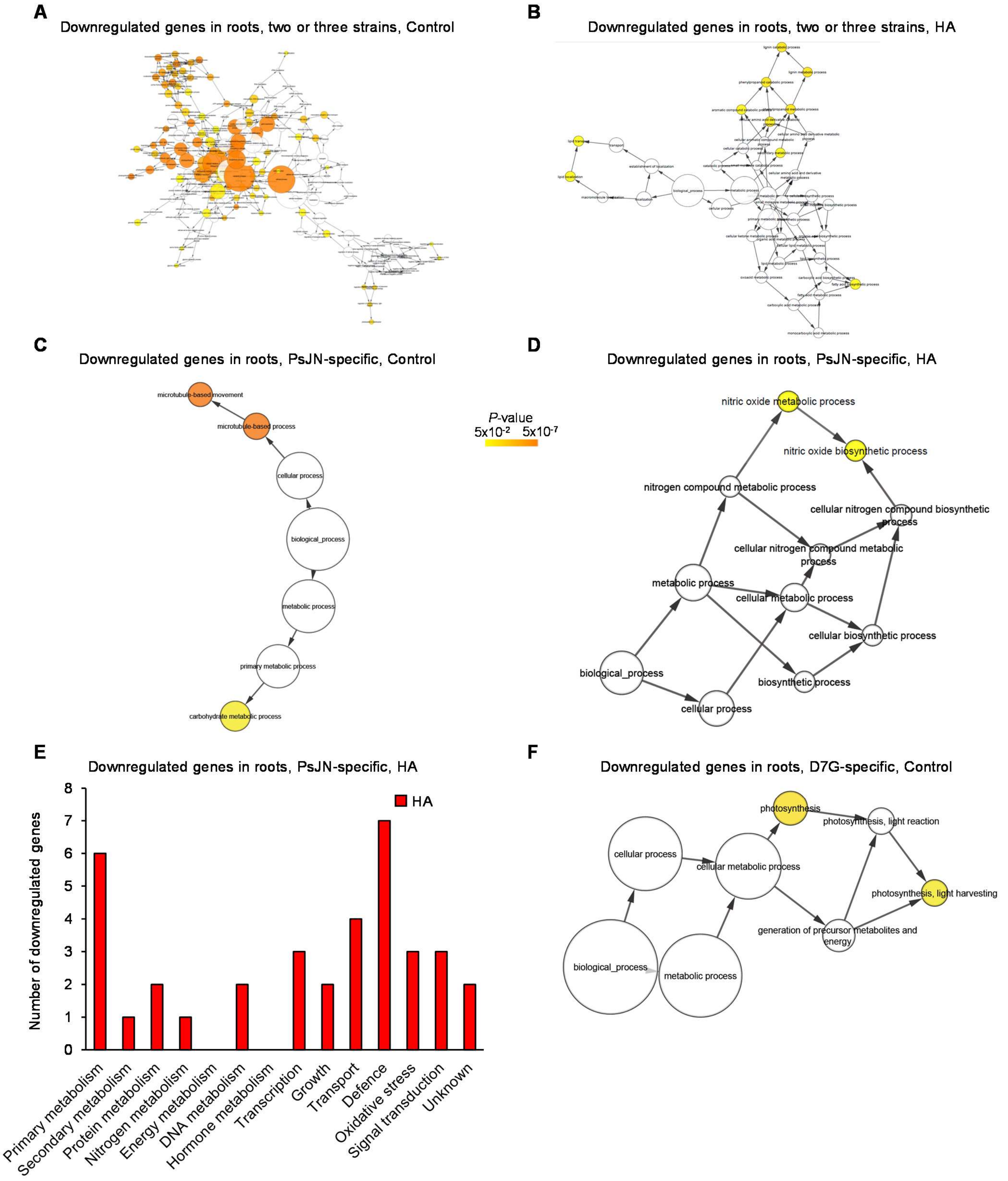

FIGURE S13 | Functional annotation of downregulated genes in tomato roots. Biological networks of significantly enriched ($P \leq 0.05$) Gene Ontology (GO) terms of downregulated genes in tomato roots in response to two or three strains among *Paraburkholderia phytofirmans* PsJN (PsJN), *Pantoea agglomerans* D7G (D7G) and *Enterobacter* sp. 32A (32A) in half-strength Hoagland with 0 mg L^{-1} (control; A) and 50 mg L^{-1}humic acid (HA; B); or specifically downregulated in response to PsJN in half-strength Hoagland it the control (C) and HA (D) condition, or to D7G in the control condition (F). The colour scale legend indicates the level of significance for enriched GO terms and white nodes indicate not significantly overrepresented categories. No significant GO enrichment was found for downregulated genes in tomato roots in response to D7G in the HA condition, as well as in response to 32A in the control and HA condition. Functional classes were assigned on the basis of the protein description of downregulated PsJN-specific genes in the HA condition (E).

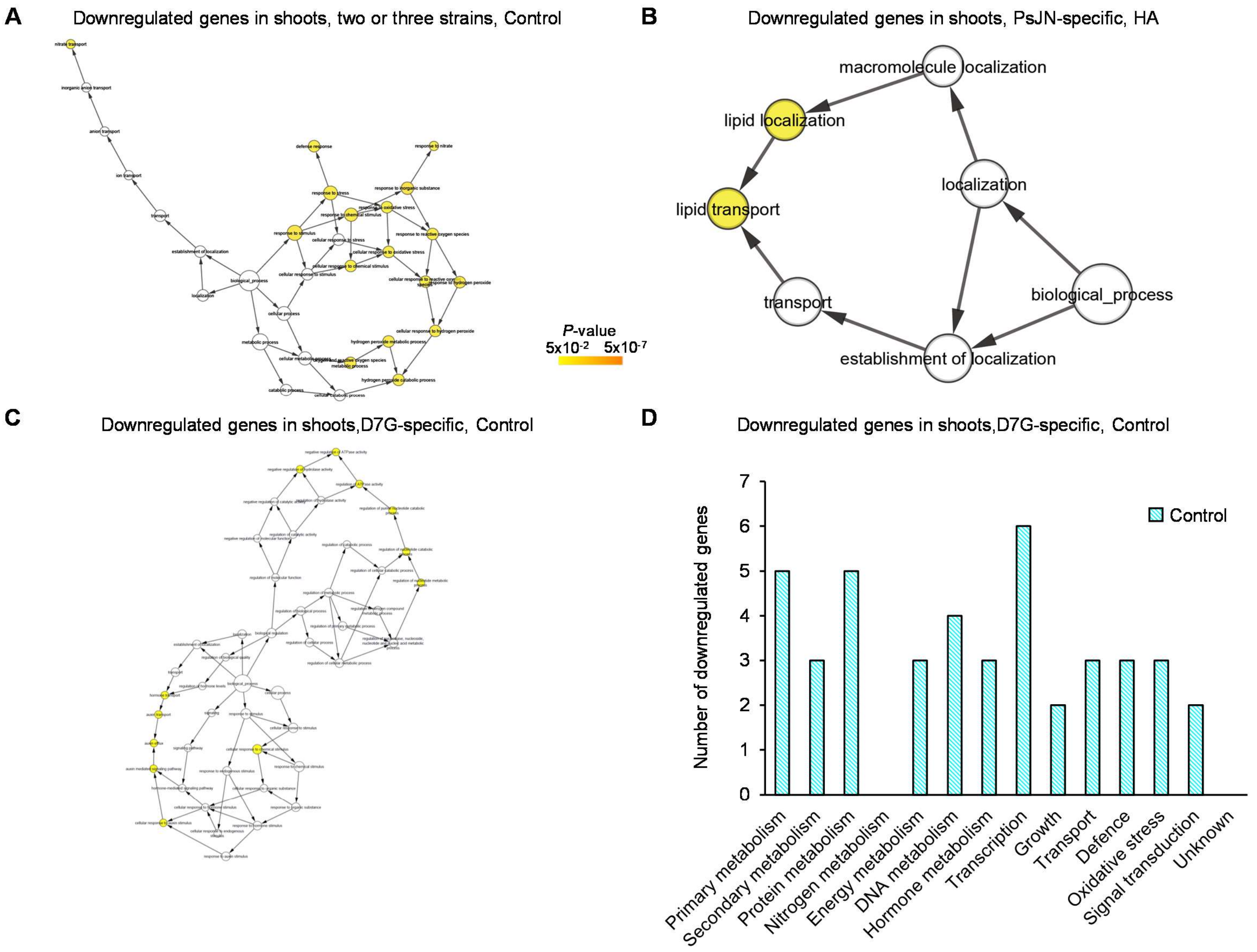

FIGURE S14 | Functional annotation of downregulated genes in tomato shoots. Biological networks of significantly enriched ($P \leq 0.05$) Gene Ontology (GO) terms of downregulated genes in tomato shoots in response to two or three strains among *Paraburkholderia phytofirmans* PsJN (PsJN), *Pantoea agglomerans* D7G (D7G) and *Enterobacter* sp. 32A (32A) in half-strength Hoagland with 0 mg L^{-1} (control; A);or specifically downregulated in response to PsJN in the HA condition (B) or D7G in the control condition (C). The colour scale legend indicates the level of significance for enriched GO terms and white nodes indicate not significantly overrepresented categories. No significant GO enrichment was found for downregulated genes in tomato shoots in response to two or three strains in the HA condition, PsJN in the control condition, D7G in the HA condition, 32A in the control and HA condition. Functional classes were assigned on the basis of the protein description of genes downregulated in tomato shoots specifically in response to D7G in the control (D).

Legends of Supplementary Excel Tables

TABLE S1 | Conversion of optical density (OD_{600} = 0.1) and colony forming units (CFU mL^{-1}) for each endophytic bacterial strain.

TABLE S2 | Primer sequences of tomato genes analyzed by quantitative real-time PCR.
Forward and reverse primer sequences of real-time quantitative RT-PCR analysis are reported for selected tomato genes. Gene abbreviation (column A), protein description (column B) from the ITAG3.2 release (https://solgenomics.net/organism/Solanum_lycopersicum/genome) and tomato gene identifier (column C). Forward (column D) and reverse (column E) primer sequences used for quantitative real-time PCR. Clustering results of differentially expressed genes (DEGs), classified as genes significantly modulated ($P \leq 0.01$ and Log_2(fold change) ≥ 1 or ≤ -1) by two or three strains (Two or three strains) or as genes modulated by only one bacterial strain (PsJN-, D7G- or 32A-specific) in the absence (control) and presence (HA) of humic acid in roots (columns G-H) and shoots (columns I-J). Mean (columns K-R) and standard error values (columns S-Z) of gene expression levels (TPM) for roots. Mean (columns AA-AH) and standard error values (columns AI-AP) of gene expression levels (TPM) for shoots. Log_2-transformed fold change values (in roots column AQ-AS and AW-AY; in shoots BC-BE and BI-BK in the control and HA condition, respectively) and *P* values (in roots column AT-AV and AZ-BB; in shoots BF-BH and BL-BN in the control and HA condition, respectively) calculated for each pairwise comparison between bacterium-inoculated and mock-inoculated samples. Log_2-transformed fold change and standard error values assessed by qPCR are reported in columns BP-CM. The tomato gene encoding ankyrin repeat domain containing protein 2 (*ARD2*) was used as constitutive genes for normalization.

TABLE S3 | RNA-Seq sequencing and mapping results for each replicate.
Tomato shoots (S) and roots (R) of mock-inoculated plants (M) and plants inoculated with *Paraburkholderia phytofirmans* PsJN (P), *Pantoea agglomerans* D7G (D) or *Enterobacter* sp. 32A (A) three days after incubation in half-strength Hoagland with 0 mg L^{-1} (control; no abbreviation in the sample code) and 50 mg L^{-1} (HA) humic acid in square dishes were analyzed in triplicate by RNA-Seq (columns A-E). Read pairs were obtained by RNA-Seq sequencing (column F) and read pairs that passed the quality check are reported in column G. Read pairs mapped to unique locations to the tomato genome and the corresponding percentage (%) of filtered read pairs are reported (columns H-I).

TABLE S4 | Expression levels of tomato genes in response to endophytic bacterial strains and humic acid.
Gene expression levels, assessed as transcripts per kilo base million (TPM), were assessed for shoots (S) and roots (R) of mock-inoculated plants (M) and plants inoculated with *Paraburkholderia phytofirmans* PsJN (P), *Pantoea agglomerans* D7G (D) or *Enterobacter* sp. 32A (A) three days after incubation in half-strength Hoagland with 0 mg L^{-1} (control; no abbreviation in the sample code) and 50 mg L^{-1} (HA) humic acid in square dishes. Tomato gene identifier (column A) and protein description (column B) from the ITAG3.2 release (https://solgenomics.net/organism/Solanum_lycopersicum/genome). Mean (columns C-J) and standard error values (columns K-R) of gene expression levels (TPM) for shoots. Mean (columns S-Z) and standard error values (columns AA-AH) of gene expression levels (TPM) for roots. Expression levels for each replicate (named from 1 to 3; columns AI-CD).

TABLE S5 | Differentially expressed genes (DEGs) identified in tomato roots three days after incubation with humic acid.
Fold change values of DEGs identified in tomato roots of mock-inoculated plants (mock) three days after incubation in half-strength Hoagland with 50 mg L^{-1} humic acid (HA condition) as compared to 0 mg L^{-1} humic acid (control). Tomato gene identifier (ITAG3.2 release, column A), Log_2-transformed fold change values (column B) and *P* values (column C). Protein description (column D) and abbreviation codes (column E) of functional classes assigned on the basis of the protein description (column N-O). Genes discussed in the manuscript are marked in bold. Gene ontology (GO) terms of the ITAG3.2 release (https://solgenomics.net/organism/Solanum_lycopersicum/genome; column F-L).

TABLE S6 | Differentially expressed genes (DEGs) identified in tomato roots three days after incubation with endophytic bacterial strains in the absence of humic acid.
Fold change values of DEGs identified in tomato roots three days after incubation with *Paraburkholderia phytofirmans* PsJN (PsJN), *Pantoea agglomerans* D7G (D7G) or *Enterobacter* sp. 32A (32A), calculated as compared to mock-inoculated plants (mock) in half-strength Hoagland with 0 mg L^{-1} humic acid (control). Tomato gene identifier (ITAG3.2 release, column A), Log_2-transformed fold change values (column B-D) and *P* values (column E-G) calculated for each pairwise comparison between bacterium-inoculated and mock-inoculated samples. Clustering results of the DEGs, classified as genes significantly modulated (in bold, $P \leq 0.01$ and Log_2 (fold change) ≥ 1 or ≤ -1) by two or three strains (Two or three strains) or as genes modulated by only one bacterial strain (PsJN-, D7G- or 32A-specific; column H). Protein description (column I) and abbreviation codes (column J) of functional classes assigned on the basis of the protein description (column S-T). Genes discussed in the manuscript are marked in bold. Gene ontology (GO) terms of the ITAG3.2 release (https://solgenomics.net/organism/Solanum_lycopersicum/genome; column K-Q).

TABLE S7 | Differentially expressed genes (DEGs) identified in tomato roots three days after incubation with endophytic bacterial strains in the presence of humic acid.
Fold change values of DEGs identified in tomato roots three days after incubation with *Paraburkholderia phytofirmans* PsJN (PsJN), *Pantoea agglomerans* D7G (D7G) or *Enterobacter* sp. 32A (32A), calculated as compared to mock-inoculated plants (mock) in half-strength Hoagland with 50 mg L^{-1} humic acid (HA). Tomato gene identifier (ITAG3.2 release, column A), Log_2-transformed fold change values (column B-D) and *P* values (column E-G) calculated for each pairwise comparison between bacterium-inoculated and mock-inoculated samples. Clustering results of the DEGs, classified as genes significantly modulated (in bold, $P \leq 0.01$ and Log_2 (fold change) ≥ 1 or ≤ -1) by two or three strains (Two or three strains) or as genes modulated by only one bacterial strain (PsJN-, D7G- or 32A-specific; column H). Protein description (column I) and abbreviation codes (column J) of functional classes assigned on the basis of the protein description (column S-T). Genes discussed in the manuscript are marked in bold. Gene ontology (GO) terms of the ITAG3.2 release (https://solgenomics.net/organism/Solanum_lycopersicum/genome; column K-Q).

TABLE S8 | Differentially expressed genes (DEGs) identified in tomato shoots three days after incubation with humic acid.
Fold change values of DEGs identified in tomato shoots of mock-inoculated plants (mock) three days after incubation in half-strength Hoagland with 50 mg L^{-1} humic acid (HA condition) as compared to 0 mg L^{-1} humic acid (control). Tomato gene identifier (ITAG3.2 release, column A), Log_2-transformed fold change values (column B) and *P* values (column C). Protein description (column D) and abbreviation codes (column E) of functional classes assigned on the basis of the protein description (column N-O). Gene ontology (GO) terms of the ITAG3.2 release (https://solgenomics.net/organism/Solanum_lycopersicum/genome; column F-L).

TABLE S9 | Differentially expressed genes (DEGs) identified in tomato shoots three days after incubation with endophytic bacterial strains in the absence of humic acid.
Fold change values of DEGs identified in tomato shoots three days after incubation with *Paraburkholderia phytofirmans* PsJN (PsJN), *Pantoea agglomerans* D7G (D7G) or *Enterobacter* sp. 32A (32A), calculated as compared to mock-inoculated plants (mock) in half-strength Hoagland with 0 mg L^{-1} humic acid (control). Tomato gene identifier (ITAG3.2 release, column A), Log_2-transformed fold change values (column B-D) and *P* values (column E-G) calculated for each pairwise comparison between bacterium-inoculated and mock-inoculated samples. Clustering results of the DEGs, classified as genes significantly modulated (in bold, $P \leq 0.01$ and Log_2 (fold change) ≥ 1 or ≤ -1) by two or three strains (Two or three strains) or as genes modulated by only one bacterial strain (PsJN-, D7G- or 32A-specific; column H). Protein description (column I) and abbreviation codes (column J) of functional classes assigned on the basis of the protein description (column S-T). Genes discussed in the manuscript are marked in bold. Gene ontology (GO) terms of the ITAG3.2 release (https://solgenomics.net/organism/Solanum_lycopersicum/genome; column K-Q).

TABLE S10 | Differentially expressed genes (DEGs) identified in tomato shoots three days after incubation with endophytic bacterial strains in the presence of humic acid.
Fold change values of DEGs identified in tomato shoots three days after incubation with *Paraburkholderia phytofirmans* PsJN (PsJN), *Pantoea agglomerans* D7G (D7G) or *Enterobacter* sp. 32A (32A), calculated as compared to mock-inoculated plants (mock) in half-strength Hoagland with 50 mg L^{-1} humic acid (HA). Tomato gene identifier (ITAG3.2 release, column A), Log_2-transformed fold change values (column B-D) and *P* values (column E-G) calculated for each pairwise comparison between bacterium-inoculated and mock-inoculated samples. Clustering results of the DEGs, classified as genes significantly modulated (in bold, $P \leq 0.01$ and Log_2 (fold change) ≥ 1 or ≤ -1) by two or three strains (Two or three strains) or as genes modulated by only one bacterial strain (PsJN-, D7G- or 32A-specific; column H). Protein description (column I) and abbreviation codes (column J) of functional classes assigned on the basis of the protein description (column S-T). Genes discussed in the manuscript are marked in bold. Gene ontology (GO) terms of the ITAG3.2 release (https://solgenomics.net/organism/Solanum_lycopersicum/genome; column K-O).
